# Increased H3K27 trimethylation contributes to cone survival in a mouse model of cone dystrophy

**DOI:** 10.1007/s00018-022-04436-6

**Published:** 2022-07-10

**Authors:** Annie L. Miller, Paula I. Fuller-Carter, Klaudija Masarini, Marijana Samardzija, Kim W. Carter, Rabab Rashwan, Xin Ru Lim, Alicia A. Brunet, Abha Chopra, Ramesh Ram, Christian Grimm, Marius Ueffing, Livia S. Carvalho, Dragana Trifunović

**Affiliations:** 1 Retinal Genomics and Therapy Group, Lions Eye Institute Ltd, 2 Verdun Street, Nedlands, WA 6009 Australia; 2 Centre for Ophthalmology and Visual Science, The University of Western Australia, 35 Stirling Hwy, Crawley, WA 6009 Australia; 3 Institute for Ophthalmic Research, Tübingen University, Elfriede-Aulhorn-Straße 7, 72076 Tübingen, Germany; 4 Lab for Retinal Cell Biology, Department of Ophthalmology, University Hospital Zürich, University of Zürich, Zurich, Switzerland; 5 Analytical Computing Solutions, Willetton, WA 6155 Australia; 6 Department of Microbiology and Immunology, Faculty of Medicine, Minia University, Minia, Egypt; 7 Institute for Immunology and Infectious Diseases, Murdoch University, Murdoch, WA Australia; 8 Department of Medicine, Vanderbilt University Medical Centre, Nashville, TN USA

**Keywords:** Inherited retinal disease, Cone photoreceptors, Achromatopsia, H3K27me3, GSK-J4, Single-cell RNA sequencing

## Abstract

Inherited retinal diseases (IRDs) are a heterogeneous group of blinding disorders, which result in dysfunction or death of the light-sensing cone and rod photoreceptors. Despite individual IRDs (Inherited retinal disease) being rare, collectively, they affect up to 1:2000 people worldwide, causing a significant socioeconomic burden, especially when cone-mediated central vision is affected. This study uses the *Pde6c*^*cpfl1*^ mouse model of achromatopsia, a cone-specific vision loss IRD (Inherited retinal disease), to investigate the potential gene-independent therapeutic benefits of a histone demethylase inhibitor GSK-J4 on cone cell survival. We investigated the effects of GSK-J4 treatment on cone cell survival in vivo and ex vivo and changes in cone-specific gene expression via single-cell RNA sequencing. A single intravitreal GSK-J4 injection led to transcriptional changes in pathways involved in mitochondrial dysfunction, endoplasmic reticulum stress, among other key epigenetic pathways, highlighting the complex interplay between methylation and acetylation in healthy and diseased cones. Furthermore, continuous administration of GSK-J4 in retinal explants increased cone survival. Our results suggest that IRD (Inherited retinal disease)-affected cones respond positively to epigenetic modulation of histones, indicating the potential of this approach in developing a broad class of novel therapies to slow cone degeneration.

**Supplementary Information:**

The online version contains supplementary material available at 10.1007/s00018-022-04436-6.

## Introduction

Inherited retinal diseases (IRDs) are a group of heterogeneous conditions that result in permanent death, dysfunction, or developmental delay of the cells in the retina, particularly the cone and rod photoreceptors [1]. Patients with IRD (Inherited retinal disease) have varying levels of visual impairment, with a large proportion eventually becoming legally blind [1]. Despite their orphan designation, they collectively affect 1:2000 people worldwide [1] and pose a heavy socioeconomic burden on patients and families with an estimated cost of £523 million to the United Kingdom alone in 2019 [2]. Polls indicate most people consider blindness the worst possible ailment they could acquire [3]. Unfortunately, limited treatments are available for IRDs (Inherited retinal disease), with most patients relying on visual aids to enhance visual acuity, and only a small subset of people with IRD (Inherited retinal disease) may be eligible for the only FDA-approved ocular gene therapy drug Luxturna^®^ or visual prosthesis [4, 5]. Over the years, considerable effort has been applied in elucidating the cell death mechanisms involved in IRD (Inherited retinal disease) pathogenesis to find novel targeting points within common cell death pathways to facilitate mutation-independent treatments. Photoreceptor degeneration in IRD (Inherited retinal disease) was classically thought to be caused by apoptosis [6, 7] but a number of recent studies revealed a novel non-apoptotic cell death pathway [8–11]. One interesting hypothesis proposed is that high cyclic guanosine monophosphate (cGMP) content present in IRD (Inherited retinal disease) photoreceptors is responsible for a cGMP (Cyclic guanosine monophosphate)-dependent activation of protein kinase G, which can lead to overactivation of histone deacetylases (HDAC) shown to be a major constituent of non-apoptotic cell death governing photoreceptor loss in IRD (Inherited retinal disease) [8, 12].

HDACs (Histone deacetylase) are ubiquitously expressed enzymes that remove acetyl groups from histones, allowing post-translational modification, chromatin structure modulation, and changes in gene expression [13]. The opposing enzymes, histone acetyltransferases (HATs), add acetyl groups, usually leading to open chromatin and increased gene expression [13]. Histone proteins may also be modified by removing or adding other chemical groups, including methyl groups. Histone demethylases (HDM) and histone methyltransferases (HMT) work in conjunction, with HDM (Histone demethylase) removing methyl groups and HMT (Histone methyltransferase) adding methyl groups. Unlike acetylation, where adding a chemical group usually leads to an increase in transcription, histone methylation results in either gene expression or repression depending on which residue of the histone protein is methylated [14]. Some methylation examples that result in gene repression include trimethylation of H3K27 and H3K9 residues, while methylation of H3K4 and H3K36 drives active gene expression [14]. The balance between these post-translational histone modifications is essential, as numerous HDAC (Histone deacetylase) and HDM (Histone demethylase) enzymes have functional interplay and are often concomitant, with histone demethylation also being reported as a secondary target of HDAC (Histone deacetylase) inhibition [15]. In the retina, HDAC (Histone deacetylase) inhibition suppresses cone photoreceptor cell loss in mouse models of two different types of IRDs (Inherited retinal disease), affecting either cones only (achromatopsia) or both rods and cones (retinitis pigmentosa, RP) [16–18]; however, there is limited knowledge on the role of histone methylation in IRD (Inherited retinal disease). One study has suggested that upregulation of specific histone methylation sites may be associated with disease in the *rd1* mouse model of RP (Retinitis pigmentosa), with global inhibition shown to delay rod photoreceptor degeneration and improve visual function [19]. A recent study showed that the administration of a lysine demethylase 1 inhibitor in the *rd10* model of RP (Retinitis pigmentosa) prevented rod death and preserved vision [20]. However, the role of histone methylation in the degeneration of cones specifically has yet to be investigated. As cone photoreceptors are affected in most types of IRD (Inherited retinal disease) and are responsible for our color, acuity and daylight vision, understanding the post-translational modifications under disease conditions could be crucial in the development of novel therapies.

The present study investigates the role of H3K27me3 in cone photoreceptor cell death in a mouse model of a human type of cone dystrophy (achromatopsia). We used the *Pde6c*^*cpfl1*^ model, which shows early onset cone loss at postnatal day (PN) 14 and peak of cell death at PN (Postnatal day) 24 due to mutations in the cone-specific *Pde6c* gene [21]. We highlight the relationship between methylation and acetylation by observing increased cone-specific H3K27me3 levels following treatment with the HDAC (Histone deacetylase) inhibitor Trichostatin A (TSA). Interestingly, the increased H3K27me3 levels in treated cones resemble the presence of H3K27me3 staining in wildtype (wt) cones. Further, we investigated the impact of GSK-J4, an H3K27 HDM (Histone demethylase) inhibitor, on cone survival in vivo and ex vivo*.* The investigation evaluated the effect of GSK-J4 in *Pde6c*^*cpfl1*^ mutant mice via histological analysis, functional testing, and single-cell RNA sequencing. We show that a single intravitreal GSK-J4 injection generated extensive alterations in gene expression, with significant enrichment of disease-related pathways, including mitochondrial dysfunction, endoplasmic reticulum stress, and epigenetic pathways. More importantly, continuous administration of GSK-J4 to *Pde6c*^*cpfl1*^ retinal explants resulted in a 31.9% increase in cone numbers compared to sham controls, indicating the potential of epigenetic modulation for the treatment of cone loss in IRD (Inherited retinal disease).

## Materials and methods

### Animals

Mice utilized in this project were housed at The University of Tübingen, University of Zürich, or The Harry Perkins Institute of Medical Research Bioresource Facility. Animals were provided with either 12/12 or 14/10 h light/dark cycle and ad libitum access to food and water, with experiments performed in accordance with the ARVO Statement for the Use of Animals in Ophthalmic and Vision Research, the regulations of the Tübingen University committee on animal protection, veterinary authorities of Kanton Zürich and The Harry Perkins Institute of Medical Research's animal ethics committee. The *Pde6c*^*cpfl1*^ mouse line (B6.CXB1-*Pde6c*^*cpfl1*^/J), originally from the Jackson Laboratory (Strain #003678) [22, 23], was used for in vivo Trichostatin A applications and for the ex vivo work. C57BL/6 J mice (Jackson Laboratory, Bar Harbor Maine, USA) served as the wildtype (wt) controls. For in vivo GSK-J4 treatment, the *Pde6c*^*cpfl1*^ line was crossbred with the Chrnb4.EGFP line (Mutant Mouse Research Resource Centre Stock #000259, herein referred to as Chrnb4.GFP) to specifically label the cones with GFP [24, 25]. Consequently, this line is referred to as *Pde6c*.GFP. Chrnb4.GFP mice on a C57BL/6 J background were used as controls for in vivo GSK-J4 work.

### Ex vivo retinal explant culture

Organotypic retinal cultures (including retinal pigment epithelium, RPE) were prepared under sterile conditions from *cpfl1* (*n* = 13) and wildtype (*n* = 4) animals, as previously described [16, 17]. PN (Postnatal day) 14 animals were euthanized by cervical dislocation or carbon dioxide asphyxiation. The eyes were enucleated and pretreated with 0.12% proteinase K (ICN Biomedicals Inc., Ohio, USA) for 15 min at 37 °C in R16, a serum-free culture medium (Gibco, Paisley, UK). Proteinase K activity was blocked by adding 10% fetal bovine serum, and the eyes were rinsed in a serum-free medium. After cornea, lens, sclera, and choroid dissection, the retina with the RPE (Retinal pigment epithelium) attached was transferred onto a culture membrane insert (Corning Life Sciences, Lowell, USA) with the RPE (Retinal pigment epithelium) facing the membrane. The membrane inserts were placed into six-well culture plates with R16 medium (Gibco) and incubated at 37 °C in a humidified 5% CO_2_ incubator. The culture medium was changed every 2 days during the 10 culturing days. Retinal explants were treated with 10 µM GSK-J4 (≥ 98% (HPLC); Sigma). GSK-J4 was dissolved in 0.2% dimethyl sulfoxide (DMSO; Sigma) and diluted in an R16 culture medium. The exact amount of DMSO was diluted in the culture medium for control explants. Explants were collected at PN (Postnatal day) 24, fixed with 4% paraformaldehyde (PFA), and cryoprotected with graded solutions of 10, 20, and 30% sucrose before embedding in tissue freezing medium (Leica Microsystems Nussloch GmbH, Nussloch, Germany).

### Intravitreal injections

Mice were anesthetized with an intraperitoneal injection of 40 mg/kg Ketamine (Ceva Animal Health Pty Ltd, Amersham, UK) and 5 mg/kg Ilium Xylazil-100 (Troy Laboratories, New South Wales, Australia) or subcutaneously with a mixture of ketamine (85 mg/kg; Parke-Davis, Berlin, Germany) and xylazine (4 mg/kg; Bayer AG, Leverkusen, Germany). Single intravitreal injections of 0.5 uL were administered at PN (Postnatal day) 14, with either 100 nM of Trichostatin A (TSA; Sigma) or 10 µM of GSK-J4 (Selleckchem, Texas, USA) administered in one eye and the contralateral eye receiving 0.0001% DMSO (diluted in 0.9% sodium chloride solution [Sigma]). GSK-J4 contained 0.0001% DMSO and was further diluted in 0.9% sodium chloride solution to make a 100 µM solution, while TSA (Trichostatin A) also contained 0.0001% DMSO with dilution to make a 100 nM solution. Assuming a 5 uL free vitreous volume [26], the final concentration of treatment was 10 nM TSA or 10 µM of GSK-J4. Anesthesia was reversed with Ilium Atipamezole (1 mg/kg; Troy Laboratories) or Antisedan (Atipamezole; 2 mg/kg; Orion Corp, Espoo, Finland).

### Immunohistochemistry

Mice were euthanized via cervical dislocation or carbon dioxide asphyxiation at PN (Postnatal day) 24, with eye orientation being marked before enucleation. Eyes were fixed in 4% PFA (Electron Microscopy Science Inc., Pennsylvania, USA) for 30 min before the cornea and iris were dissected. Eyes were fixed for another 30 min in 4% PFA before being placed into a sucrose gradient (Sigma) for cryoprotection. After 24 h, the lens was removed, and the posterior eyecup was frozen in Tissue-Tek^®^ O.C.T™ Compound (Sakura Finetek, California, USA) before being cryosectioned on the sagittal plane. Retinal sections were rehydrated with 1X phosphate-buffered saline (PBS) for 20 min at room temperature (RT) before blocking solution was applied for 1 h at RT. Blocking solution contained 0.5% Triton X 100 (ThermoFisher, Massachusetts, USA), 1% Bovine Serum Albumin (Bovogen Biologicals Pty Ltd., Victoria, Australia), and 10% Normal Goat Serum (Sigma), diluted in 1X PBS. Primary and secondary antibodies were applied to detect antigens in samples (complete list of antibodies in Table S1). Following application of primary and secondary antibodies, slides were incubated for 5 min with DAPI (4',6-diamidino-2-phenylindole, 0.5 ug/mL in 1X PBS) and were mounted using fluorescence mounting medium (Dako). Negative controls were included in each batch by omitting the primary antibody.

### Microscopy and cell counting

Images from retinal explants were captured using Z-stacks on a Zeiss Axio Imager Z1 ApoTome Microscope using the 20X air objective. The number of cones was quantified on 6–16 different positions, from 3 to 6 retinal cross-sections obtained from different positions within a retinal explant. In vivo images were taken on the Nikon Ti-E inverted motorized microscopy with Nikon A1Si spectral detector confocal system, with Z-stacks obtained on the 20X air objective. Quantification was performed as previously described [18], where each biological replicate had two sections imaged, and each section had an image taken in four positions, relative to the optic nerve: + 10° (superior, central), + 80° (superior, peripheral), − 10° (inferior, central) and − 80° (inferior, peripheral) positioning. The operator was blinded, and the numbers of GFP-positive cells (cones) were counted manually. The length of the outer nuclear layer (ONL) was manually measured using NIS-C Elements software. Cone quantification data were expressed as the number of cones/mm. Calculation of H3K9 and H3K27me3 positive cones was performed by counting a population of cones and determining the percentage of positive cells. Outer nuclear layer thickness was determined by manually counting the number of photoreceptor rows in nine different areas of retinal cryosections. To determine the placement of cone photoreceptors, we analyzed the position of cone nuclei relative to the ONL (Outer nuclear layer) thickness. The relative position of every individual cone in the image to the outer plexiform layer (OPL) was calculated by dividing the basal and central position of the cone soma from the OPL (Outer plexiform layer) by the total thickness of the ONL (Outer nuclear layer). A value closer to 0 indicates the cone is closer to the OPL (Outer plexiform layer) while a value closer to 1 indicates the position is closer to the outer limiting membrane. The average central displacement of wt (Wildtype) retinae were used to define the displacement threshold of 0.8, where lower values indicate cone displacement. All qualitative histology presented is representative images of staining throughout the tissue and from at least three biological replicates.

### Electroretinography (ERG)

Retinal function in uninjected, sham-injected, and GSK-J4 injected mice was assessed at PN (Postnatal day)24 in the *Pde6c*.GFP line. Both full-field flash scotopic and photopic recordings were performed on the Celeris full-field ERG (Electroretinography) system (Diagnosys LLC, Massachusetts, USA). Mice were dark-adapted overnight, and handled under dim red light for scotopic readings. Mice were anesthetized with an intraperitoneal injection of 80 mg/kg Ketamine (Ceva Animal Health Pty Ltd) and 10 mg/kg Ilium Xylazil-100 (Troy Laboratories), and pupils were dilated by application of a drop of 1% tropicamide to the cornea (Alcon, Western Australia). A drop of 2% hypromellose (HUB pharmaceuticals LLC, California, USA) was also applied to the cornea and eye electrodes to ensure moisture was retained and to act as a contact fluid. Animals were kept warm throughout the procedure with the in-built platform heater on the Celeris system. For scotopic readings, single-flash intensities were obtained through 1 ms flashes with intensities of 0.01, 0.1, 0.3, 1, 3, 10, and 25 cd.s.m^−2^. The time between consecutive flashes was 10 s, while the stimulus was repeated four times at 0.10 Hz. 60 s recovery time between different flash intensities was allowed. Following scotopic readings, mice were light-adapted for 10 min at 30 cd.s.m^−2^. A series of flashes on a background of 30 cd.m^−2^ were performed at 2 Hz and at intensities of 3 and 10 cd.s.m^−2^. The time between consecutive flashes was 0.5 s, while the stimulus was repeated 32 times. Flicker ERG (Electroretinography) responses were recorded at a pulse frequency of 10 and 30 Hz, with a background of 30 cd.m^−2^ and a pulse intensity of 3 cd.s.m^−2^. Analysis of a- and b-waves was performed on the Espion V6 software (Diagnosys LLC).

### Cell dissociation and single-cell sorting of cones

Fresh retinae were dissected before being placed in activated papain/DNase (1:20 solution; Worthington Biochemicals, New Jersey, USA) and incubated at 37 °C for 45 min on a shaker. After incubation, the solution was gently triturated before spinning at 1500 rpm for 5 min. The supernatant was discarded before the cell pellet was resuspended in Earle’s balanced salt solution (EBSS; Worthington Biochemicals) that contained 9.5%v/v Ovomucoid Inhibitor and 5%v/v DNase (Worthington Biochemicals). This resuspended solution was incubated at 37 °C for 10 min and then re-spun at 1500 rpm for 5 min. The supernatant was discarded, and the cell pellet was resuspended in EBSS and 10%v/v DNase. Samples were incubated with eBioscience™ Fixable Viability Dye eFluor™ 660 (Invitrogen, California, USA) on ice and in the dark for 30 min. Samples were then washed in fluorescent-activated cell sorting (FACS) buffer that contained 2% heat-inactivated fetal calf serum (Fisher Biotec, Wembley, Western Australia), 1 mM EDTA (Invitrogen), and 1X PBS. Cells were pelleted at 1400 × rpm for 5 min, the supernatant discarded and resuspended in FACS (Fluorescence-activated cell sorting) buffer. Cell sorting was performed on a BD FACS (Fluorescence-activated cell sorting) Melody Cell Sorter, with single GFP-positive cells collected into 96-well plates containing 3 uL of lysis buffer and ribonuclease inhibitor (one cell/well). Sorted plates were stored at − 80 °C until processed.

### Single-cell RNA sequencing and bioinformatics

Sample preparation (RNA extraction, library preparation) and sequencing was performed at the Institute for Immunology and Infectious Diseases (Murdoch University, Perth, Western Australia) using the method described in Wanjalla et al. 2021 [27], which is an adapted version of the SMARTseq2 and MARS-seq approaches [28, 29]. Briefly, the assay utilized uniquely tagged primers for reverse transcription and template switching with a pre-amplification step to increase the yield and transcript length of the single-cell cDNA library. During the initial reverse transcription step, cDNA was tagged with well-specific barcodes coupled with a unique molecular identifier (UMI) to allow for multiplexing and increased sample throughput. Samples were then pooled and amplified using the KAPA HiFi HotStart ReadyMix (Roche, Basel, Switzerland), as per the manufacturer’s instructions. UMI (Unique molecular identifier)s enabled quantitation of individual gene expression levels within single cells, thereby reducing technical variability and bias introduced during the amplification step [30–32]. Purified amplicons from the 5' and 3' were pooled to equimolar amounts, and indexed libraries were created for sequencing using Truseq adapters and quantified using the Kapa universal qPCR library quantification kit (Kapa Biosystems Inc., MA, USA). Samples were sequenced on an Illumina Novaseq using a 2 × 100 paired-end chemistry kit (Illumina Inc., CA, USA), as per the manufacturer’s instructions. Reads were quality-filtered and passed through a demultiplexing tool to assign reads to individual wells and to the 3' end and 5' end. Reads for the individual single cells were demultiplexed using plate-ID (30 nt), and cell barcode (6 nt). The reads were further demultiplexed as either 3' or 5' using primer sequence (30 nt), and the reminder 45 nt sequences were aligned to the GRCh38 Mus-Musculus reference genome (Ensembl rel. 97) using the CLC Genomics Workbench (CLC Bio) (v.20, QIAGEN Bioinformatics). An eight-nucleotide UMI (Unique molecular identifier) tag and mapping coordinates were used to remove PCR-duplicate reads. Gene-specific read counts were calculated using RSubread:featureCount using Gencode M17 (2018) annotations, and the 3' and 5' counts were summed. Genes with > 0 counts in fewer than three cells and cells that either contained less than 200 genes or more than 5% mitochondrial content were filtered out. Downstream analyses (normalization, principal component analysis, differential expression, and visualization) were performed in Seurat v.2.3.4 R package [33]. A significance threshold of unadjusted *P* < 0.01 and absolute log-fold > 0.3 was used to identify differentially expressed genes (DEGs), with batch correction applied using the Combat approach [34]. The Enrichr suite of enrichment analysis tools [35] was used to perform gene ontology analysis of biological processes, with redundant terms trimmed using Revigo [36] (unadjusted *P* < 0.01). In silico analysis of processed ChIP-seq (Chromatin immunoprecipitation sequencing) data (from the ENCODE Histone Modifications 2015 database accessed via Enrichr) was used to identify methylation peaks near DEG (Differentially expressed gene)s. Ingenuity Pathway Analysis (Qiagen) was used for canonical pathway and network analysis. To identify and define any subpopulations, cells were clustered on the basis of their expression profiles using Seurat to perform unsupervised UMAP (Uniform manifold approximation and projection) analysis, along with Clustering Trees software to identify the optimal number of clusters [37]. The Seurat FindAllMarkers function was used to identify gene expression markers that were more highly expressed in each cluster, relative to all other clusters, indicating transcriptionally related cells regardless of genotype (a marker gene defined as being > 0.3 log-fold higher (unadjusted *P* < 0.01) than the mean expression value in the other sub-clusters, and with a detectable expression in > 25% of all cells from the corresponding sub-cluster). All raw data have been deposited into the Gene Expression Omnibus (GEO) repository GSE195895.

### Western blot

Retinae were homogenized in RIPA-lysis buffer. Following cell debris removal by centrifugation, supernatants were precipitated using ice-cold acetone. Proteins were resuspended in NuPAGE™ LDS Sample Buffer containing reducing agent (Invitrogen, California, USA), separated on 12% SDS-PAGE gels and transferred on PVFD membranes. Membranes were exposed overnight to the primary antibodies (Acetyl-Histone H4 (Lys8) Antibody, Cell Signaling, Massachusetts, USA; β-actin, Cell Signaling, Massachusetts, USA) and for 1 h to HRP-conjugated anti-rabbit secondary antibody (1:2.000, Cell Signaling, Massachusetts, USA). Immunoreactivity was visualized with Pierce™ ECL Western Blotting Substrate (Thermo Fisher Scientific, Massachusetts, USA) and detected with FusionFX instrument (Vilber Lourmat, France).

### Statistical analysis

Statistical analysis was performed using the PRISM software (GraphPad; San Diego, USA) and Excel (Microsoft; Washington, USA). Either *t*-test or ANOVA was used to analyze result significance (*P* < 0.05, except for single-cell RNA sequencing where *P* < 0.01).

## Results

### HDAC inhibition increases H3K27 trimethylation in *Pde6c*^*cpfl1*^ cones

**Fig. 1:**
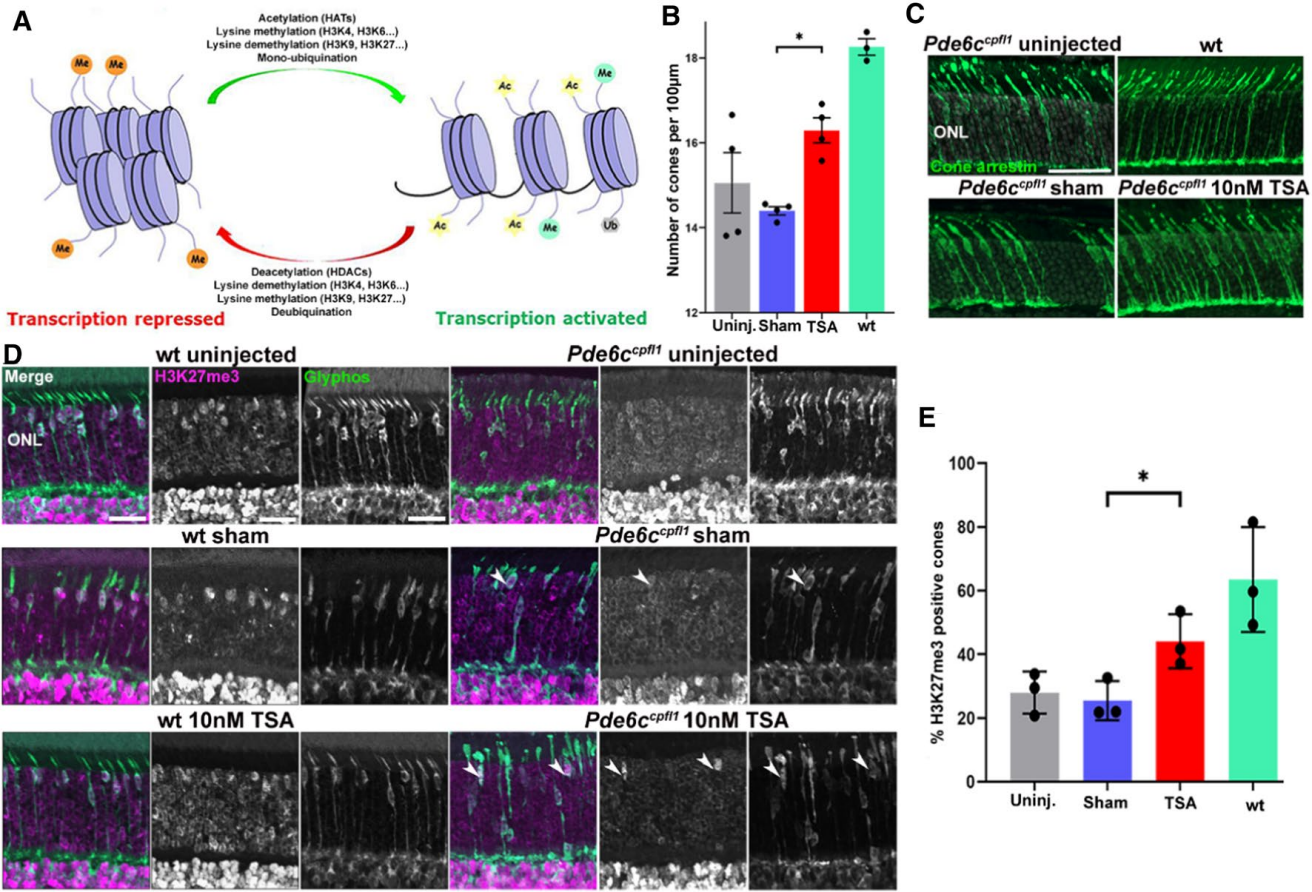
In vivo intravitreal delivery of Trichostatin A (TSA) at PN (Postnatal day)14 increases H3K27 trimethylation and cone numbers at PN (Postnatal day) 24 in the *Pde6c*^*cpfl1*^ mouse model of cone dystrophy.** A** The balance and interplay between repressed and activated transcription. **B**, **C** A single intravitreal injection of Trichostatin A (TSA) led to significant cone (green) retention in *Pde6c*^*cpfl1*^ mice compared to sham-injected controls. One-way ANOVA, *n* = 4, *P < 0.01. Scale bar 50 µm. ONL, outer nuclear layer. **D** Uninjected and sham-injected *Pde6c*^*cpfl1*^ mice showed scarce H3K27me3 methylation (magenta) colabeling with Glyphos (glycogen phosphorylase in green). However, the TSA (Trichostatin A)-treated *Pde6c*^*cpfl1*^ cones displaced H3K27 trimethylation pattern similar to wt (Wildtype), wt (Wildtype) sham and wt (Wildtype) TSA (Trichostatin A)-treated cones. Arrows indicate cells that show co-localization of H3K27me3 and Glyphos in *Pde6c*^*cpfl1*^ mice. Scale bar 20 µm. **E** Quantification of the percentage of H3K27me3 positive cones showed a significant increase in H3K27me3 localization in the cone photoreceptors, in TSA (Trichostatin A)-treated retinae compared to sham-injected controls. Welch’s *T*-test, *n* = 3, *P < 0.01

Interaction between complex and diverse epigenetic modifications results in active transcription (via the activity of histone acetyltransferases, lysine methylases, lysine demethylases, and ubiquitin) or repressed transcription (histone deacetylases, lysine methylases, lysine demethylases, and de-ubiquitinating enzymes; Fig. 1A). To study this relationship specifically in cone photoreceptor degeneration, we have investigated the effect of in vivo delivery of the pan-HDAC (Histone deacetylase) inhibitor Trichostatin A (TSA) on the methylation status on histone H3 lysine residues in *Pde6c*^*cpfl1*^ mice. A single intravitreal injection of 10 nM TSA (Trichostatin A) at PN (Postnatal day)14 led to a significant increase in cone survival up to PN (Postnatal day)30 compared to sham treatment (Fig. 1B, C; One-way ANOVA ** < 0.01, *n* = 4). This finding additionally emphasizes the beneficial effect of HDAC (Histone deacetylase) inhibition on photoreceptor cell survival in multiple models of IRD (Inherited retinal disease) [15–17]. We assessed the levels of H3K27 methylation in wt (Wildtype) and *Pde6c*^*cpfl1*^ controls (uninjected and sham-injected) and TSA (Trichostatin A)-treated cones by co-staining with H3K27me3-specific and cone-specific glycogen phosphorylase (Glyphos) antibodies [16, 38] (Fig. 1D). H3K27 trimethylation was detected in the cones of uninjected, sham-injected and TSA (Trichostatin A)-treated wt (Wildtype) mice, while in uninjected and sham-injected *Pde6c*^*cpfl1*^ mice, staining of H3K27me3 was seldomly observed in the cones (Fig. 1D). Treatment with 10 nM TSA (Trichostatin A) resulted in a partial restoration of H3K27me3 levels with 44% of the TSA (Trichostatin A)-treated cones expressing H3K27me3 in comparison to 25% H3K27me3 positive cones in sham-injected retinae (Fig. 1E; Welch's T-test, * < 0.05, n = 3). We investigated the effect of TSA (Trichostatin A) on another epigenetic marker H4K8 acetylation (H4K8ac), with immunostaining patterns being unchanged (Fig. S1A). H3K9me3, which has similar gene silencing properties as H3K27me3, resulted in a similar number of H3K9me3 positive cones after the treatment (Fig. S1B, C). This was validated by Western blot of the whole retina, showing a similar protein abundance after 10 nM of TSA (Trichostatin A) was administered (Fig. S1D). As the changes to the H3K27 trimethylation status in the cones of *Pde6c*^*cpfl1*^ mice appeared to be markedly downregulated, we further investigated if administration of a drug that inhibits demethylation at H3K27 sites would be a suitable neuroprotective treatment to prevent cone loss.

### The histone demethylase inhibitor GSK-J4 induces global transcriptional changes in the Pde6c^cpfl1^ cones

**Fig. 2:**
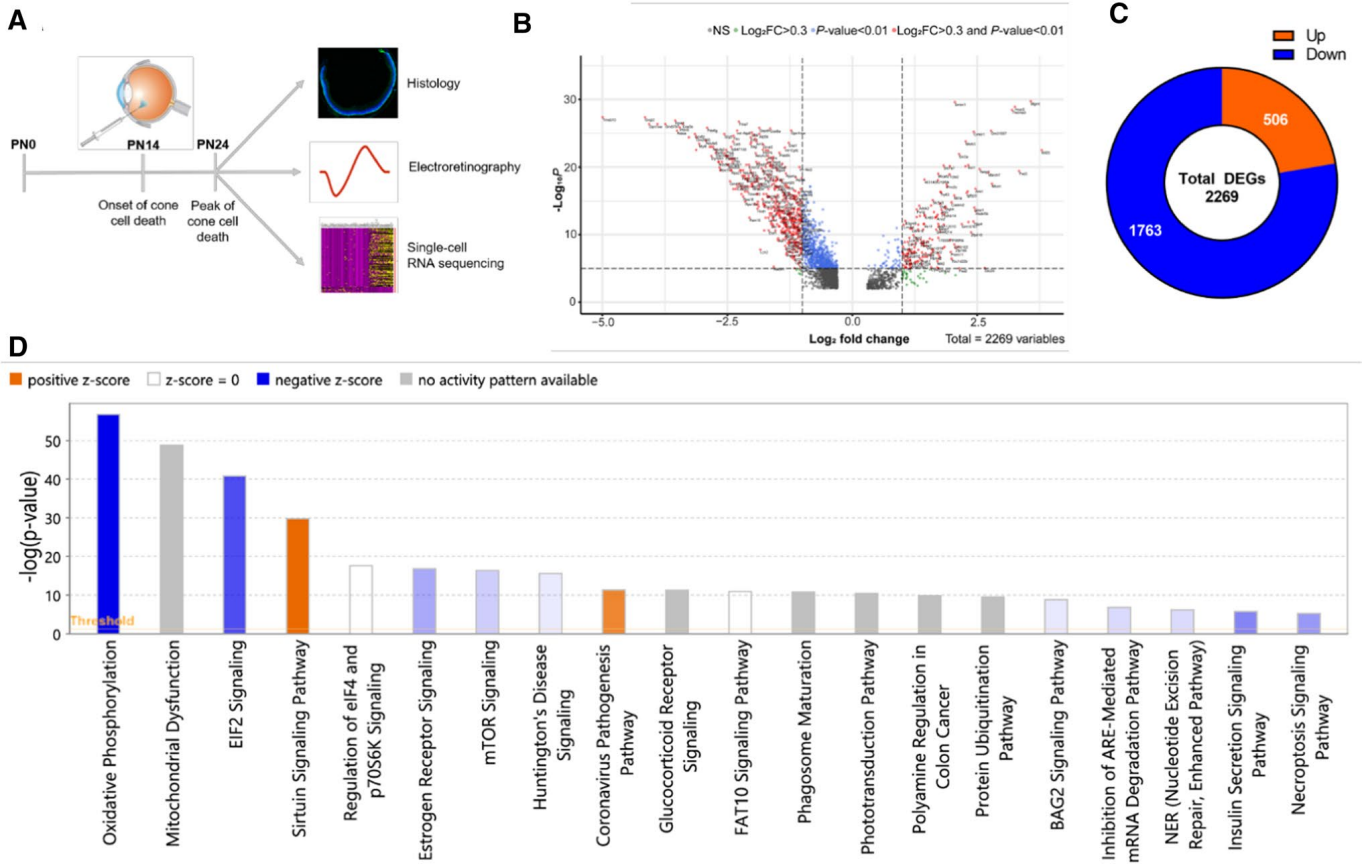
Administration of a single intravitreal injection of GSK-J4 at the onset of cone cell death in *Pde6c*.GFP mice induced global transcriptional changes. **A** Schematic overview of the experimental design of in vivo GSK-J4 intravitreal delivery at the onset of cell death (PN (Postnatal day) 14) and sample collection at the peak of cell death (PN (Postnatal day) 24). Eyes were collected for histological analysis, electroretinography, and single-cell RNA sequencing of the cones. **B** Volcano plot of differentially expressed genes after GSK-J4 treatment, based on their –Log_10_ *P-value* and Log_2_ fold change. Genes in red were analyzed further, as they were considered biologically relevant, using benchmarks of a *P-value* < 0.01 and a fold change greater than 0.3 or less than -0.3. **C** Pie chart showing the number of genes that were upregulated (< 25%) and downregulated (> 75%) in our dataset. **D** Ingenuity pathway analysis bar graph, depicting the most enriched canonical pathways following the GSK-J4 administration (i.e., mitochondrial function and dysfunction, endoplasmic reticulum stress, epigenetic pathways, among others). The Z score represents the overall activation (yellow) or repression (blue) of each pathway, dependent on the direction of gene expression within the pathway. Intravitreal injection image available under license: Creative Commons Attribution-NonCommerical 3.0 Unported at https://creativecommons.org/licenses/by-nc/3.0/; https://researchgate.net/figure/Intravitreal-injection-Note-Intravitreal-injection-of-drug-is-recently-used-for_fig2_235729464

We have previously shown that inhibition of histone deacetylases in a mouse model of RP (Retinitis pigmentosa) results in transcriptional changes of numerous cone-specific genes [18]. To assess to which extent direct modulation of histone methylation is associated with changes in cone-specific transcription profiles, we performed a single intravitreal injection in the *Pde6c*^*cpfl1*^ mice with the Jumonji H3K27me2/me3 demethylase inhibitor GSK-J4 at the onset of cone degeneration, PN (Postnatal day) 14 (Fig. 2A) [21]. At PN (Postnatal day) 24, the peak of cone death, we tested cone function and gene expression to assess the effects of the treatment (Fig. 2A). We used our previously validated transgenic *Pde6c*.GFP mouse, which is phenotypically the same as the *Pde6c*^*cpfl1*^ mouse. Our *Pde6c*.GFP mouse carries the same mutations in the *Pde6c* gene as the *Pde6c*^*cpfl1*^ alongside a copy of the e*GFP* gene downstream of the promoter for the *Chrnb4* gene, driving GFP expression exclusively in the cones. This allows for easy quantification and cell sorting of cone photoreceptors [24]. Using GFP fluorescence, we employed fluorescent-activated cell sort (FACS Fluorescence-activated cell sorting) of GSK-J4 treated and uninjected retinae to isolate cones for single-cell RNA sequencing. After processing and quality control, a total of 85 untreated cones and 71 GSK-J4-treated cones were analyzed. With a significance of unadjusted *P* < 0.01 and an absolute log-fold change greater than 0.3, we identified 2269 differentially expressed genes (DEG (Differentially expressed gene)s), with more than 75% of them being downregulated (Fig. 2B, C; full list in Table S2).

We evaluated upregulated and downregulated genes after the GSK-J4 treatment and utilized the Enrichr gene ontology (GO) database to determine the functional association of these genes. The most upregulated DEG (Differentially expressed gene)s included genes associated with action potentials (*Scn11a*, *Cacna1b*, *Cacna1a*, *Scn3a*, *Cacna1h*, *Scn2b*, and *Kcnd2*) and nucleotide-excision repair (*Parp1*, *Ercc4*, *Rpa2*, *Cul4b*, and *Rfc2*) along with a wide variety of other biological processes. On the other hand, we observed a significant downregulation in various mitochondrial processes, including ones influenced by ATP synthase presence (*Atp5a1*, *Atp5b*, *Atp5c1*, *Atp5d*, *Atp5e*, *Atp5f1*, *Atp5g1*, *Atp5g2*, *Atp5g3*, *Atp5h*, *Atp5j*, *Atp5j2*, *Atp5k*, *Atp5l*, *Atp5o1*, *mt-Atp6*, and *mt-Atp8*), cytochrome C function (*Cox4i1*, *Cox5a*, *Cox5b*, *Cox6a1*, *Cox6b1*, *Cox6b2*, *Cox6c*, *Cox7a2*, *Cox7a2l*, *Cox7b*, *Cox7c*, *Cox8a*, *Cox14*, and *Cox19*), RNA polymerase II subunits (*Polr2c*, *Polr2d*, *Polr2e*, *Polr2f*, *Polr2g*, *Polr2h*, *Polr2j*, *Polr2k*, *Polr2l*, and *Polr2m*) and other protein targeting and translational genes (full list of genes and GO (Gene ontology) in Table S2-S4). We also observed GSK-J4 induced expression changes in several histone modifiers, including histone (*Kmt1d*, *Kmt2d*, *Kmt5c*, and *Prmt2*) and non-histone (*Eef1akmt*) methyltransferases, demethylases (*Kdm7c*, *Prdm11*), acetyltransferases (*Atf2*, *Atf4*), and deacetylases (*Hdac4*, *Sirt2*). All were downregulated with the exception of *Prdm11* and *Hdac4.* Two components of the MLL4-COMPASS complex (*Dpy30*, *Ash2l*) were also downregulated, including the H3K27me2/3 demethylase *Kdm6a* and H3K4me1/2/3 methyltransferase *Kmt2d* (Tables 1 and S2). Furthermore, results from Enrichr ENCODE Epigenetics database revealed that H3K27me3 was associated with the promoter region of 55 of our DEG (Differentially expressed gene)s, including genes involved in apoptosis (5) and post-translational modification (6) (Table S5).

**Table 1:** Histone modifying genes affected by the GSK-J4 treatment

Histone modification	Gene symbol
Methyltransferases (Kmt)	*Kmt1d*, *Kmt2d*, *Kmt5c*, *Eef1akmt*
Demethylases (Kdm)	*Kdm7c*
Acetyltransferases (Hat)	*Atf2*, *Atf4*
Deacetylases (Hdac)	*Sirt2*, ***Hdac4***
Other	*Ashl2*, *Dpy30*, *Prmt2*, ***Prdm11***

The single-cell RNA sequencing data suggest that all genes displayed were downregulated, except *Hdac4* and *Prdm11*, which were upregulated (bolded)

**Fig. 3:**
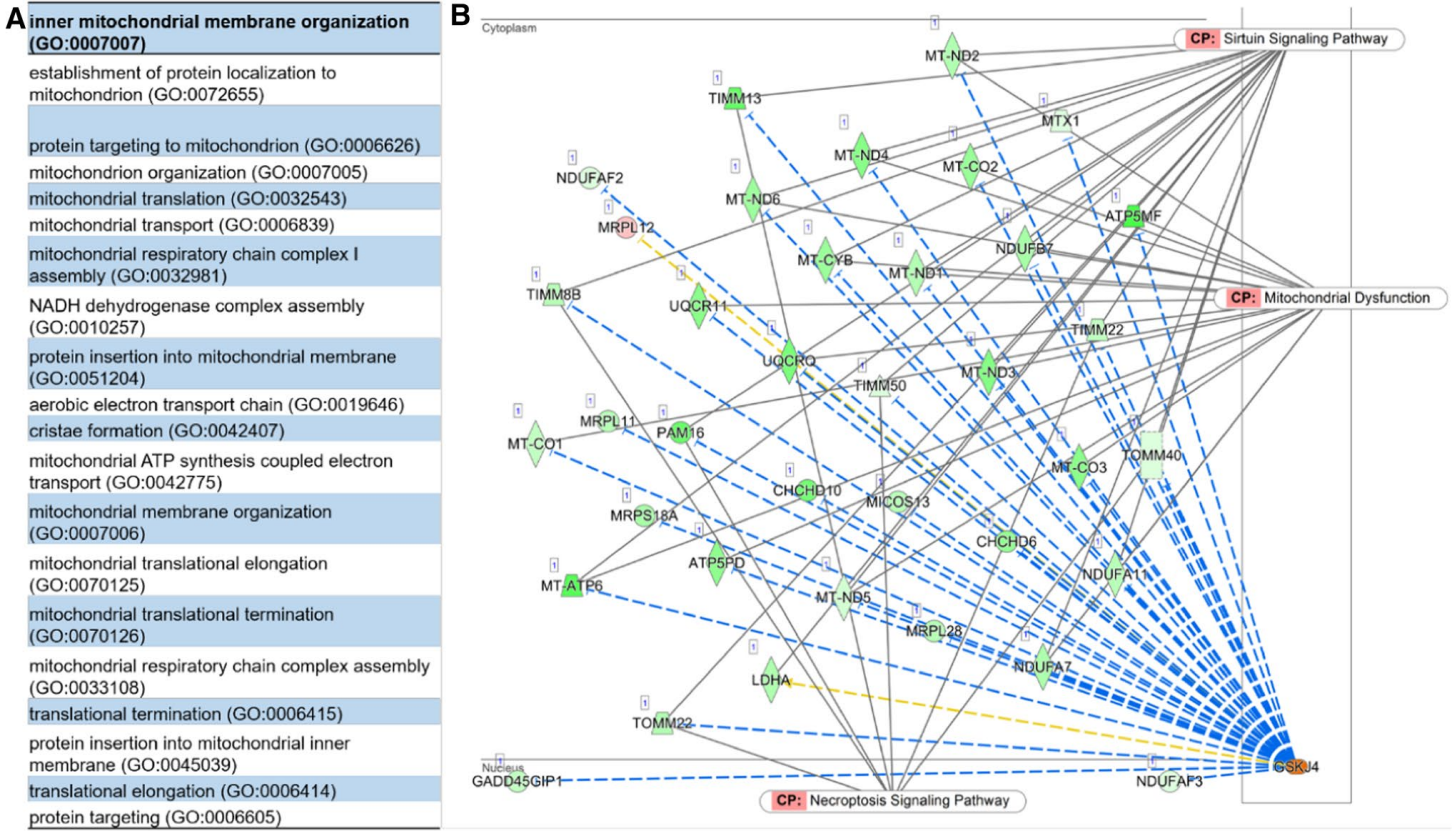
A complex interaction between different pathways following the GSK-J4 treatment. **A** The most enriched biological processes after GSK-J4 treatment, showing substantial enrichment of mitochondrial function and organization, and translation. Gene ontology analysis was performed using Enrichr, with redundant terms trimmed using Revigo (unadjusted *P* < 0.01). **B** Network visualization of the downstream targets of GSK-J4 with the canonical pathways overlaid. Many of these genes are involved in the mitochondrial dysfunction pathway, while others show involvement in sirtuin and necroptosis signaling pathways. Schematic downloaded from QIAGEN Ingenuity Pathway Analysis. Green downregulated; red upregulated. Blue leads to inhibition, and yellow indicates inconsistent findings into a downstream state of a molecule

Canonical pathway analysis was performed using the Ingenuity Pathway Analysis tool (IPA; Qiagen), with the top 20 enriched pathways shown in Fig. 2D. More specifically, within the top three pathways, GSK-J4-treated cones showed downregulation of the oxidative phosphorylation, mitochondrial dysfunction, and EIF2 signaling pathways (Fig. S2 and S3; Full list of canonical pathways in Table S6). Further pathway analysis highlighted mitochondrial function, organization, and protein translation as key biological processes associated with GSK-J4 treatment (Fig. 3A). Downstream gene targets of GSK-J4 included translocases of mitochondrial membranes (*Timm13*, *Timm8b*, *Timm22*, *Timm50*, *Pam16*, *Tomm40*, and *Tomm22*), NADH dehydrogenases (*mt-Nd1*, *mt-Nd2*, *mt-Nd3*, *mt-Nd4*, *mt-Nd5*, and *mt-Nd6*), NADH-ubiquinone oxioreductases (*Ndufaf2*, *Ndufaf3*, *Ndufa7*, *Ndufb7*, and *Ndufa11*), cytochrome-related molecules (*mt-Co1*, *mt-Co2*, *mt-Co3*, *mt-Cyb*, *Uqcr11*, and *Uqcrq*), among others (Fig. 3B).

### Gene expression clustering and downstream targets following GSK-J4 treatment

**Fig. 4:**
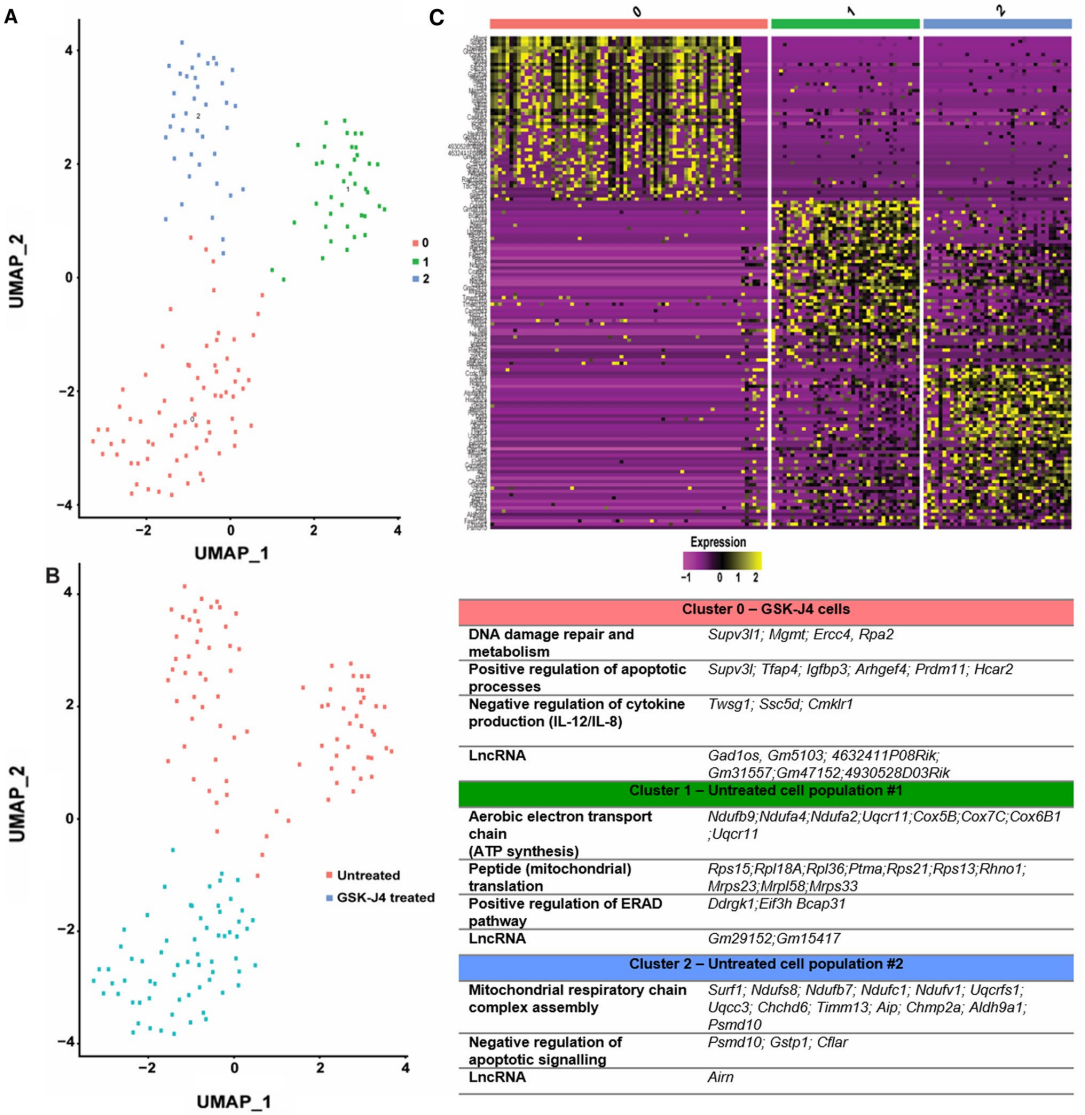
The GSK-J4 treatment-induced changes in cone photoreceptor clustering and gene expression.** A**, **B** Clustering analysis of cones visualized on a UMAP (uniform manifold approximation projection) plot revealed three clusters, with cluster 0 comprising the GSK-J4-treated cones, while the cones from uninjected *Pde6c.*GFP mice showed two different cell clusters, clusters 1 and 2. **C** Heat map of the top 50 most expressed genes that characterize each cluster, with the table showing the enriched genes and gene ontology (GO) biological processes associated with each cluster. Gene expression is scaled and presented as Log2-transformed fold change

The single-cell approach allowed us to examine the cellular heterogeneity of cones in response to GSK-J4 treatment and to identify genetic markers that may be used to discriminate cone subpopulations. An unsupervised uniform manifold approximation and projection (UMAP Uniform manifold approximation and projection) plot was used to visualize the Seurat clustering analysis, which plots transcriptionally similar cells using a weighted k-nearest neighbor method [39] (Fig. 4A). Clusters were identified based on their gene expression profile (top 50 genes for each cluster are shown in Table S7), defined as being > 0.3 log-fold higher than the mean expression value in the other sub-clusters, and with a detectable expression in > 25% of all cells from the corresponding sub-cluster. Annotation of the clusters revealed that cluster 0 predominantly contained GSK-J4-treated cones, while clusters 1 and 2 comprised primarily of untreated cones (Fig. 4B). Interestingly, untreated cone cells formed two distinct clusters while GSK-J4-treated cones formed one. Genes from the cluster heat map, representing the top 50 genes from each cluster (selected by average log-fold change; Fig. 4C), were used for GO (Gene ontology) analysis to identify characteristics of each cluster (see Table S6 for the complete list of genes and GO (Gene ontology)). Biological processes associated with cluster 0 focused on DNA repair and damage response and positive regulation of apoptosis. Genes involved with oxidative phosphorylation (complexes I and IV of the aerobic electron transport chain) and activation of endoplasmic reticulum stress response defined cluster 1. Cluster 2 was enriched for respiratory chain function (complexes I and III) and negative regulation of apoptosis. Interestingly, the expression of other known histone modifiers, long non-coding RNA (lncRNA), was also a descriptor of each cluster, with cluster 0 containing six lncRNA (Long non-coding RNA)s in the top 50 genes (*Gad1os*, *Gm5103*, *4632411P08Rik*, *Gm31557*, *Gm47152*, and *4930528D03Rik*), compared to two lncRNA (Long non-coding RNA)s for cluster 1 (*Gm29152*, *Gm15417*) and one lncRNA (Long non-coding RNA) for cluster 2 (*Airn*).

### In vivo assessment of the histone demethylase inhibitor GSK-J4 on cone photoreceptor survival and function

**Fig. 5:**
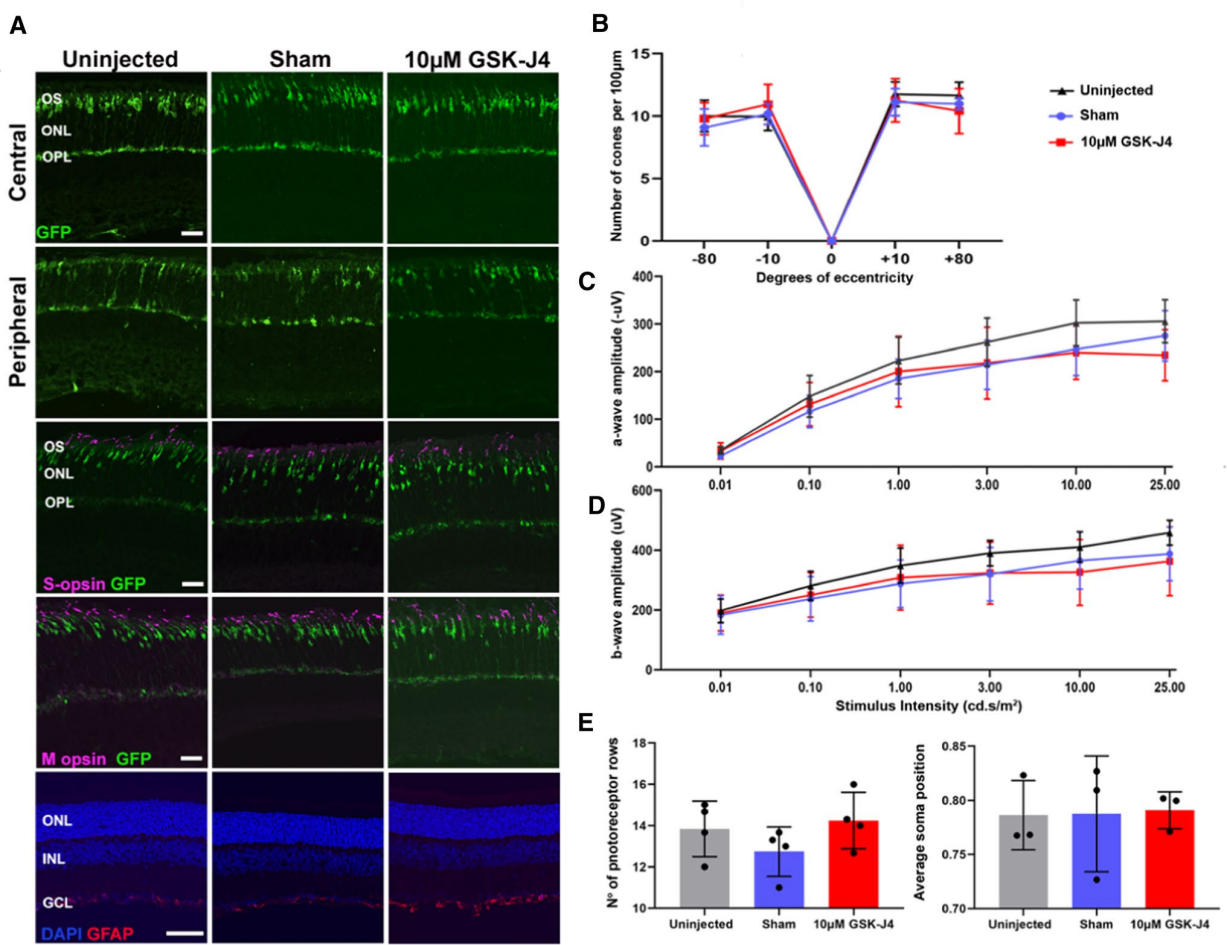
An intravitreal injection of GSK-J4 in *Pde6c*.GFP mice at PN (Postnatal day) 14 does not affect morphology, cone numbers, protein localization or retinal function at PN (Postnatal day) 24. **A** Representative central and peripheral retinal images from the *Pde6c.*GFP mouse line with GFP-positive cones (green) showing general morphology is similar between uninjected, sham-injected and GSK-J4-treated mice. S- and M-opsin (magenta) localization appears consistent between treated and uninjected groups. GFAP (Glial fibrillary acidic protein) expression (red) is similar between uninjected and sham-injected mice, with a mild increase in expression after treatment with GSK-J4. Nuclei are stained with DAPI (blue). Scale bars 20 µm. OS, outer segment. ONL, outer nuclear layer. OPL, outer plexiform layer. INL, inner nuclear layer. GCL, ganglion cell layer. GFAP (Glial fibrillary acidic protein), glial fibrillary protein. GFP, green fluorescent protein. **B** Quantification of cone numbers per 100 µm ONL (Outer nuclear layer) length across the retina indicates that cone numbers do not differ between *Pde6c.*GFP uninjected, sham-injected or GSK-J4-treated retinae. Locations of the retina imaged were central superior (corresponding to + 10° eccentricity from the optic nerve labeled with 0), peripheral superior (+ 80°), central inferior (− 10°) and peripheral inferior (− 80°). Two-way ANOVA, *n* = 7, *P* > 0.05. **C** Dark-adapted (scotopic) amplitude of the a-wave at different stimulus intensities, revealed no difference between uninjected, sham and GSK-J4-treated *Pde6c.*GFP mice. Two-way ANOVA, *n* = 4, *P* > 0.05. **D** Amplitude of the scotopic b-wave at six different stimulus intensities, showing no significant difference after treatment with GSK-J4. Two-way ANOVA, *n* = 4, *P* > 0.05. **E** Evaluation of the ONL (Outer nuclear layer) thickness after treatment with a single intravitreal GSK-J4 injection showed no difference. One-way ANOVA, *n* = 4, *P* > 0.05. **F** Average central displacement of the cone photoreceptor soma was not changed after treatment. One-way ANOVA, *n* = 4, *P* > 0.05

While treatment with GSK-J4 in *Pde6c.*GFP mice induced robust changes in cone-specific transcription profiles, we did not observe a difference in cone numbers compared to controls (Fig. 5B; 2-way ANOVA, *P* > 0.05, *n* = 7). We validated this with IHC staining, with all groups showing a similar number of cone cells, cone morphology, opsin localization, and glial fibrillary acidic protein expression as a marker for Müller glia activation (Fig. 5A). We also performed photopic (cone-function) and scotopic (rod-function) ERG (Electroretinography) recordings to assess any functional changes to the retina after treatment with GSK-J4. We did not observe a significant difference at any stimulus intensity in photopic or scotopic ERG (Electroretinography) between *Pde6c.*GFP groups in either a- or b-wave amplitude, suggesting the treatment did not have any deleterious effects on photoreceptor function (Fig. 5C, D; photopic data not shown; 2-way ANOVA, *P* > 0.05, *n* = 4). Assessment of ONL (Outer nuclear layer) thickness and cone displacement revealed no change (Fig. 5E, F; One-way ANOVA, *P* > 0.05, n = 4). We noted a slight increase in Iba1-positive microglia in *Pde6c.*GFP retinae, but with no activation or migration to the ONL (Outer nuclear layer), after GSK-J4 administration (Fig. S4A). Furthermore, we assessed the effect of PN (Postnatal day) 14 10 μM GSK-J4 administration on PN (Postnatal day) 24 wt (Wildtype) retinae. Assessment of GFAP (Glial fibrillary acidic protein) and Iba1 staining in wt (Wildtype) controls revealed no increase in GFAP (Glial fibrillary acidic protein) levels, and a very slight increase in Iba1-positive microglia, with no activation or migration observed (Fig. S4B, C). ERG (Electroretinography) recordings of wt (Wildtype) controls injected with either sham or GSK-J4 no changes in any stimulus intensity in both scotopic and photopic recordings, indicating that GSK-J4 administration in the wt (Wildtype) retinae does not negatively impact retinal function (Fig. S4D–G).

### Continuous GSK-J4 treatment led to cone protection ex vivo

**Fig. 6:**
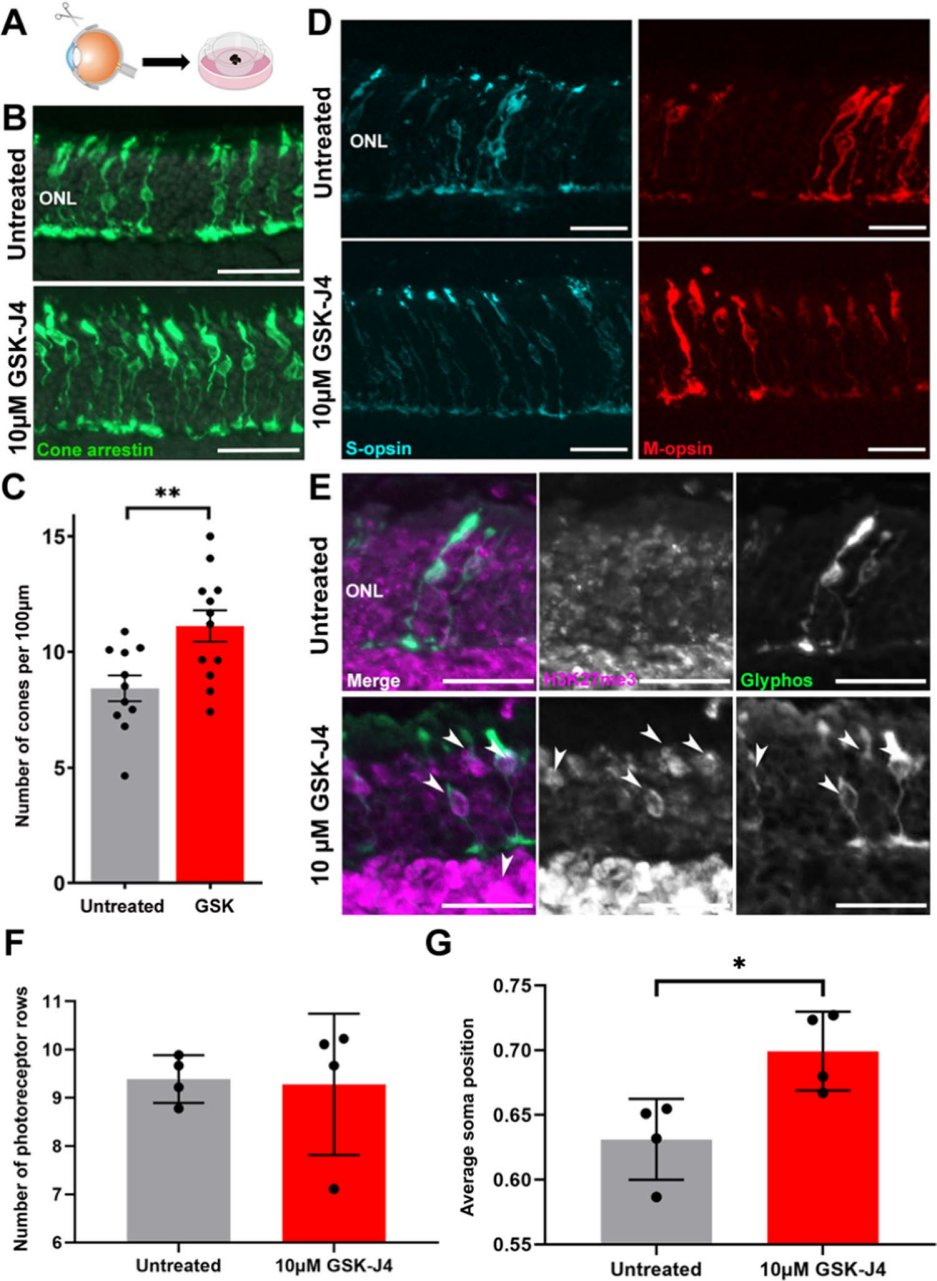
Continuous delivery of GSK-J4 to *Pde6c*^*cpfl1*^ retinal explants for 10 days allowed significant retention of cone photoreceptors, improved morphology, and localization of M- and S-opsins.** A** Schematic diagram showing the process of dissecting the retina from PN (Postnatal day) 14 *Pde6c*^*cpfl1*^ mice before growing retinal explants on culture membranes. Retinal explants were either sham-treated or treated with 10 µM of GSK-J4 daily for 10 days before collection at PN (Postnatal day) 24. **B**, **C** Representative cone arrestin (green) immunostaining and quantification of cone numbers in *Pde6c*^*cpfl1*^ retinal explants show significant protection of GSK-J4-treated cones compared to untreated explants*.* Welch’s *T*-test, *n* = 11 untreated, *n* = 12 treated, **P < 0.01. Scale bar 50 µm. ONL, outer nuclear layer. **D** M-opsin (red) and S-opsin (cyan) immunostaining showed an improvement of protein localization in the cones after treatment with GSK-J4. Scale bar 20 µm. ONL, outer nuclear layer. **E** An increase of H3K27me3 (magenta) staining was observed in *Pde6c*^*cpfl1*^ explants that had received GSK-J4 treatment (indicated by arrows) but not in untreated explants. Scale bar 20 µm. ONL, outer nuclear layer. Glyphos, glycogen phosphorylase. **F** The number of photoreceptor rows was unchanged between untreated and GSK-J4-treated groups. Welch’s *T*-test, *n* = 4, *P* > 0.05. **G** The average soma position of cones relative to the outer limiting membrane was significantly improved after administration of 10 µM GSK-J4. Welch’s *T*-test, *n* = 4, *P ≤ 0.05

Although a single dose of GSK-J4 in vivo did not result in an increased cone survival at PN (Postnatal day) 24, our single-cell RNA sequencing data suggested a beneficial effect of the drug via downregulation of disease-related pathways, including endoplasmic reticulum stress and mitochondrial dysfunction pathways. A single intravitreal injection may not have been sufficient to affect cone survival 10 days post-injection due to the short half-life of GSK-J4. We assumed GSK-J4 followed extensive intravitreal clearance, shown for similar small lipophilic molecules [16, 40]. Hence, we tested the effect of continuous GSK-J4 treatment on cone survival. For this, we used ex vivo retinal explants of PN (Postnatal day) 14 *Pde6c*^*cpfl1*^ mice, allowing the continuous presence of GSK-J4 treatment for 10 days (Fig. 6A). Quantification on cones in GSK-J4-treated explants showed a 31.9% increase in cone survival when compared to sham controls (Fig. 6B, C; Unpaired *T*-test with Welch’s correction, **P < 0.01, *n* = 11 sham, *n* = 12 treated). We also observed an improved localization of M- and S-opsin and increased H3K27me3 staining in the cones after the treatment (Fig. 6D, E). We evaluated ONL (Outer nuclear layer) thickness after treatment, with no significant difference in the number of photoreceptor rows between treatment groups (Fig. 6F; Unpaired *T*-test with Welch’s correction, *P* > 0.05, *n* = 4). Finally, we assessed the differences in cone displacement revealing a decrease in cone soma displacement after treatment with GSK-J4 (Fig. 6G; Unpaired *T*-test with Welch’s correction, *P < 0.05, *n* = 4).

## Discussion

*Pde6c*^*cpfl1*^* cones have reduced expression of H3K27me3*, *which is partially restored by TSA (Trichostatin A) administration* Methylation and other key epigenetic markers are important in photoreceptor development, differentiation, fate determination, and function [41, 42]. Mature rods have a hypomethylated DNA epigenome, with a closed chromatin state when compared to cones, indicating an overall repressed genetic environment [42]. Specifically, rods contain high levels of the repressive H3K9me3 in the nucleus, while cones show the presence of the active chromatin marker H3K4me3 [43, 44]. These differences between chromatin condensation and methylation marks give rise to the distinct identity of rods and cones [43]. The cone-specific methylation reported here adds weight to the hypothesis of Ueno and colleagues [44], which proposes that mature cones lose H3K27ac and H3K4me1 but gain DNA methylcytosines. Furthermore, this study is the first to confirm that cones specifically require H3K27met3. Consequent changes in these patterns may lead to harmful effects with genes being incorrectly repressed or expressed. Indeed, a previous study in the *rd1* model of RP (Retinitis pigmentosa) showed upregulation of the repressive methylation site H3K27me3 in rods whereby inhibition of histone methylation delayed rod degeneration [19]. Similarly, inhibition of lysine demethylase 1, which specifically demethylates H3K4me1/2 and H3K9me1/2, blocks rod degeneration in *rd10* mice [20]. Contrary to rods, our study shows that wt (Wildtype) cones have high levels of H3K27me3, which is diminished in degenerating *Pde6c* mutant cones. This finding suggests that while increased levels of H3K27me3 levels appear to be deleterious in rods, the absence of H3K27 trimethylation may be associated with cone degeneration. As the chromatin in mouse rods is already hypercondensed, the upregulation of repressive methylation marks such as H3K27me3 push nuclei towards pyknosis and subsequent cell death [45], while the same disease mechanism may not happen in mouse cones due to their different natural chromatin condensation. The role of H3K27me3 and their corresponding demethylases is complex, with studies showing the role of *Kdm6b* (a demethylase of H3K27me3) in the development of the earlier born retinal cells; amacrine, horizontal, retinal ganglion cells as well as rod bipolar cells [46]. Raeisossadati et al. 2019 also showed that injection of GSK-J1 in PN (Postnatal day) 0 mice identified an integral role for *Kdm6b* in processes such as cell proliferation, apoptosis, differentiation, senescence and cellular reprogramming [47].

Aberrant histone methylation status has been discussed as a possible pathological feature in various neurodegenerative diseases, including Alzheimer’s, Huntington’s, amyotrophic lateral sclerosis, and IRD (Inherited retinal disease) [19, 48]. A particular focus point is HDAC (Histone deacetylase) inhibition, classically utilized in cancer treatment, which has recently shown a neuroprotective capacity in many diseases, including IRD (Inherited retinal disease) [49]. Several studies show the complex interactions between many epigenetic pathways, including that histone methylation is a secondary target of HDAC (Histone deacetylase) inhibitors [50]. Our study shows that treatment with the HDAC (Histone deacetylase) inhibitor TSA (Trichostatin A) provides significant retention of cone numbers [16, 17], while also causing an increase in H3K27me3 in the cones of the *Pde6c*^*cpfl1*^ mutant mouse. Other groups have observed this phenomenon, with Halsall et al. 2015 showing in lymphoblastoid cells that treatment with other pan-HDAC (Histone deacetylase) inhibitors SAHA and valproic acid, resulted in an upregulation of H3K27me3 at transcription start sites which are thought to promote cell survival through adaptive mechanisms via minimization of protein hyperacetylation, slowing growth and re-balancing patterns of gene expression [51]. It is possible that the increase in H3K27me3 we observed after TSA (Trichostatin A) administration treatment is related to TSA (Trichostatin A)’s ability to decondense chromatin, making these sites more available for antibody detection. However, studies using other non-histological methods, such as MALDI-MS (Matrix-assisted laser desorption/ionization mass spectrometry), LC–MS/MS (Liquid chromatography tandem mass spectrometry) or ChiPseq analyses, have been able to directly quantify and show changing levels of H3K27me3 and H3K9me3 after TSA (Trichostatin A) administration [51–53]. Although TSA (Trichostatin A) provided an increase in H3K27me3, our approach for this study was to take one step further and ask if directly inhibiting H3K27me3 demethylation via GSK-J4 administration could be used to promote cone survival pathways.

*The effect of GSK-J4 on epigenetic modifiers* GSK-J4 was initially developed as a selective inhibitor of the H3K27me2/3 demethylases *Kdm6a* and *Kdm6b* but was later found to inhibit multiple members of the Jumonji-C domain-containing family, including the Kdm5 subfamily, which is responsible for the demethylation of active H3K4me1/2/3 markers [54]. As the methylation state of both H3K4 and H3K27 can be protective in neurodegeneration [20, 55], it indicates a role for GSK-J4 in concomitantly inhibiting both *Kdm6* and *Kdm5* subfamilies and suggest its neuroprotective action may involve the activity of both repressive H3K27me and active H3K4me.

While we did not directly identify specific demethylases, our data points to the involvement of *Kdm6a*, both as a means of regulating H3K27me3 deposition and as a critical part of a protein group that regulates H3K4 methylation. In the MLL-COMPASS protein complex, KDM6A associates with the H3K4me1/2/3 methyltransferase, KMT2D, along with a core group of accessory proteins (WDR5, RbBP5, ASH2L, DPY30; the WRAD complex) [56]*.* Our single-cell RNA sequencing data showed that *Kmt2d*, along with two core protein genes (*Dpy30* and *Ash2l*), were downregulated after GSK-J4 treatment. This complex is involved in guiding RNA polymerase II to engage transcription [57], and we show downregulation of ten out of twelve RNA polymerase II subunits, suggesting a possible mechanism by which overall transcription-associated processes are decreased.

*Treatment of GSK-J4 leads to significant alterations in pathways associated with cell survival and mitochondrial function* After treatment with GSK-J4, we saw a substantial shift in the gene expression profile of single cones when visualized to investigate their relationship with one another. Untreated *Pde6c.*GFP cells clustered into two distinct subpopulations, with vastly different associated biological processes. Cluster 1 (untreated group 1) showed upregulation of the endoplasmic-reticulum-associated protein degradation pathway, which suggests increased cell death via misfolded or non-functional proteins [58]. Misfolded protein accumulation has been observed in other forms of , with a study in P23H Rho models showing that this accumulation is a part of the disease progression [59]. On the other hand, cluster 2 (untreated group 2) showed enrichment of the mitochondrial assembly chain and a decrease in apoptosis. The dichotomy of cone cell scattering and associated biological processes suggests that at least at PN (Postnatal day) 24, *Pde6c* mutant cones are at two different stages of the disease. One population of cells shows increased levels of cell death via protective mechanisms, indicating these cells might be in advanced stages of degeneration, while the other population has a decrease in apoptosis, meaning they may be in the early stages of the disease.

Interestingly, after treatment with GSK-J4, we observed one uniform population of cell clustering. The associated biological processes after GSK-J4 administration included DNA repair and damage responses and, surprisingly, positive regulation of apoptosis. A possible explanation for the increase in apoptosis after GSK-J4 treatment is that some cells that cannot be restored by DNA repair mechanisms may undergo apoptosis, while salvageable cells are repaired [60]. We also observed a decrease in pro-inflammatory cytokines involved in IRD (Inherited retinal disease) pathogenesis [61]. While GSK-J4 may facilitate apoptosis of cells that have gone past the point of no return, at the same time, it may have beneficial effects to still viable cells by reducing inflammatory conditions in the retina and enabling cell repair.

Overall, *Pde6c* mutant cones showed significant changes in several pathways associated with epigenetic modification, cell survival, and mitochondrial function as a consequence of the GSK-J4 treatment, with the most enriched canonical pathways being oxidative phosphorylation, mitochondrial dysfunction and EIF2 signaling, all of which were downregulated. Abnormal mitochondria function and eye disease have been linked numerous times. Both inherited, and age-related retinal diseases show abnormal mitochondria function and the build-up of reactive oxygen species (as reviewed by Lefevere et al. 2017) [62]. We observed the downregulation of many mitochondrial genes and processes, including ones related to cytochrome *c*. Cytochrome *c* is integral to healthy mitochondrial processes; however, overactivation has been extensively linked to cell death via activation of caspases [63]. Caspase related cell death’s involvement in IRD (Inherited retinal disease) is widely debated. While Venkatesh et al. 2017 indicated that Casp-7 is not involved in two separate models of RP (Retinitis pigmentosa), other studies have shown varying roles of caspases and apoptotic cell death in IRDs (Inherited retinal disease) [64, 65]. An intriguing study by Huang et al. 2004 showed that the mitochondria play an important role in light-induced retinal degeneration models, suggesting that downregulation of three proteins; ATP synthase subunit-6, cytochrome *c* oxidase-III, and NADH dehydrogenase-3, was neuroprotective [66]. We observed a downregulation of their corresponding genes, *mt-Atp6*, *mt-CO3*, and *mt-Nd3*, suggesting the involvement of this pathway after treatment with GSK-J4. We also observed downregulation of superoxide dismutases (SODs), involved predominantly in the mitochondrial dysfunction pathway. The role of SOD (Superoxide dismutase)s in RP (Retinitis pigmentosa) has been extensively studied, with previous studies showing induction of cell death by increased expression of SOD (Superoxide dismutase)2 [67], as well as data showing downregulation of SOD (Superoxide dismutase)1 in the *rd10* mouse model [68]. Following GSK-J4 treatment, genes involved in the CHOP cell death pathway (part of the EIF2 signaling pathway) appear significantly repressed. Activation of the CHOP pathway can lead to cell death via long-term endoplasmic reticulum stress, causing induction of pro-apoptotic genes and suppression of anti-apoptotic proteins [69]. Other models of IRD (Inherited retinal disease) have indicated an involvement of this pathway in cellular degeneration, with the *rd16* model of Leber’s congenital amaurosis showing an increase in the components of the CHOP pathway, leading to increased cell death [70]. Previous studies into the mechanism of action of GSK-J4 in an acute myeloid leukemia model indicate that GSK-J4 induces cell cycle arrest and apoptosis in cancer cells via the CHOP pathway [71]. This concept is intriguing, in that regulation of this pathway in cancer cells induces cell death, while in our model of cone degeneration it may contribute to neurodegeneration.

*Continuous GSK-J4 treatment has a significant effect on cone survival in Pde6c*^*cpfl1*^* retinal explants* While the data obtained by single-cell RNA sequencing suggested protective effects of GSK-J4 via the regulation of aberrant methylation and demethylation and indicated potential anti-apoptotic properties, we did not see a significant increase in cone survival 10 days after a single intravitreal injection of GSK-J4. Since short-term exposure to GSK-J4 treatment was able to affect key pathways potentially involved in the pathogenesis of IRD (Inherited retinal disease), we evaluated the effect of continuous GSK-J4 treatment by assessing cone survival in ex vivo retinal explants after 10 days of treatment. The observed ~ 32% increase in cone numbers compared to sham controls and improvement in cone migration [16, 21] suggests that GSK-J4 treatment may be beneficial when administered regularly or through drug delivery systems that allow continuous administration. While achromatopsia involves the specific loss of cones, we investigated the effect GSK-J4 may be having on the rods by assessing the ONL (Outer nuclear layer) thickness and the scotopic ERG (Electroretinography) response due to the integral relationship between rods and cones. The communication between rods and cones may be mediated by molecules such as microRNAs and connexins and may involve dopamine and cannabinoid signaling [72–75]. Gap junctions have been suggested to contribute to cone cell death by the transfer of toxic molecules from rods to healthy cones in complex diseases such as age-related macular degeneration, glaucoma and diabetic retinopathy. The relevance in IRDs (Inherited retinal disease) like RP (Retinitis pigmentosa), however, is still up for debate [76]. A study by Kranz et al. 2013 suggested that deletion of Connexin36 did not alter the characteristic secondary cone death in the Rho and *rd1* models of RP (Retinitis pigmentosa) [77, 78]. Despite its importance, our data suggest that the rod–cone relationship does not play a significant role after administration with GSK-J4, as we did not observe changes in the ONL (Outer nuclear layer) thickness or scotopic ERG (Electroretinography) readings, and our treatment did not influence connexin expression in cones. Since GSK-J4 can induce changes at multiple levels, it may represent a broader treatment option that could be used in multiple IRD (Inherited retinal disease) forms or in complex cone-loss diseases like age-related macular degeneration. Further studies should be conducted to assess the effectiveness of GSK-J4 in other models of IRD (Inherited retinal disease) while utilizing a continual drug delivery system, such as nanoparticle-based delivery. It also remains to be investigated if combining neuroprotective agents, such as GSK-J4, with gene therapies can provide improved recovery of visual function compared to gene therapy alone. While human trials of the first FDA-approved retinal gene therapy, Luxturna, showed significant visual improvement, it was reported that retinal degeneration still occurs despite the treatment [79]. Therefore, a combinatorial treatment strategy may be a way forward in such situations, allowing for the retention of cells and preventing the eventual degradation of the patient’s vision due to cellular loss [79].

### Supplementary Information

Below is the link to the electronic supplementary material.

Supplementary file1 (XLSX 259 KB)

Supplementary file2 (DOCX 42910 KB)

## Data Availability

The datasets generated and analyzed during the current study are available in the Gene Expression Omnibus repository and are accessible through GEO series accession number GSE195895. Reviewers may use the private token mhszgiaofdczfyh at https://www.ncbi.nlm.nih.gov/geo/query/acc.cgi?acc=GSE195895.

## Abbreviations

IRD: Inherited retinal disease
cGMP: Cyclic guanosine monophosphate
HDAC: Histone deacetylase
HAT: Histone acetyltransferase
HDM: Histone demethylase
HMT: Histone methyltransferase
RP: Retinitis pigmentosa
PN: Postnatal day
TSA: Trichostatin A
wt: Wildtype
RPE: Retinal pigment epithelium
ONL: Outer nuclear layer
OPL: Outer plexiform layer
ERG: Electroretinography
FACS: Fluorescence-activated cell sorting
UMI: Unique molecular identifier
DEG: Differentially expressed gene
UMAP: Uniform manifold approximation and projection
GO: Gene ontology
lncRNA: Long noncoding RNA
GFAP: Glial fibrillary acidic protein
MALDI-MS: Matrix-assisted laser desorption/ionization mass spectrometry
LC-MS/MS: Liquid chromatography tandem mass spectrometry
ChIP-seq: Chromatin immunoprecipitation sequencing
SOD: Superoxide dismutase
