# Supplementary material for: Increased H3K27 trimethylation contributes to cone survival in a mouse model of cone dystrophy
Source: Cell Mol Life Sci. 2022 Jul 10;79(8):409. doi: 10.1007/s00018-022-04436-6 (PMC9271452; doi:10.1007/s00018-022-04436-6)
Supplement: Supplementary file 2 — Supplementary file2 (DOCX 42910 KB) [file 18_2022_4436_MOESM2_ESM.docx]

**Title:** Increased H3K27 trimethylation contributes to cone survival in a mouse model of cone dystrophy

Journal: Cellular and Molecular Life Sciences

Authors and affiliations: Annie L. Miller^1,2^, Paula I. Fuller-Carter^1^, Klaudija Masarini^3^, Marijana Samardzija^4^, Kim W. Carter^5^, Rabab Rashwan^1,6^, Xin Ru Lim^1,2^, Alicia A. Brunet^1,2^, Abha Chopra^7,8^, Ramesh Ram^7^, Christian Grimm^4^, Marius Ueffing^3^, Livia S. Carvalho^1, 2^*, Dragana Trifunović^3^*

^1^ Retinal Genomics and Therapy Group, Lions Eye Institute Ltd, 2 Verdun Street, Nedlands, 6009 WA, Australia

^2^ Centre for Ophthalmology and Visual Science, The University of Western Australia, 35 Stirling Hwy, Crawley, 6009 WA, Australia

^3^ Institute for Ophthalmic Research, Tübingen University, Elfriede-Aulhorn-Straße 7, 72076 Tübingen, Germany

^4^ Lab for Retinal Cell Biology, Department of Ophthalmology, University Hospital Zürich, University of Zürich, Zürich, Switzerland

^5^ Analytical Computing Solutions, Willetton, 6155, WA, Australia

^6^ Department of Microbiology and Immunology, Faculty of Medicine, Minia University, Minia, Egypt

^7^ Institute for Immunology and Infectious Diseases, Murdoch University, Murdoch, WA, Australia

^8^ Department of Medicine, Vanderbilt University Medical Centre, Nashville, TN, United States

*** Correspondence:** LSC liviacarvalho@lei.org.au; DT draganatri@yahoo.com

**Table S1.** **Summary of primary and secondary antibodies used for immunohistochemistry**

| **Antibody** | **Host species** | **Supplier** | **Cat no.** | **Working dilution** | **Incubation** |
| --- | --- | --- | --- | --- | --- |
| GFP Polyclonal Antibody, AlexaFluor 488 | Rabbit | Invitrogen | A-21311 | 1:500 | 2 hours room temperature |
| Glial Fibrillary Acidic Protein Polyclonal Antibody | Rabbit | DAKO | Z0334 | 1:500 | 4^o^C overnight |
| Anti Iba1 | Rabbit | Wako | 019-19741 | 1:500 | 4^o^C overnight |
| Tri-Methyl-Histone H3 (Lys27) | Rabbit | Cell Signalling | 9733S | 1:200 | 4^o^C overnight |
| H3K27me3 Polyclonal Antibody | Rabbit | Invitrogen | PA5-31817 | 1:200 | 4^o^C overnight |
| Histone H3 [Trimethyl Lys9] Antibody | Mouse | Novus Biologicals | NBP1-30141SS | 1:200 | 4^o^C overnight |
| Acetyl-Histone H4 (Lys8) Antibody | Rabbit | Cell Signalling | 2594 | 1;200 | 4^o^C overnight |
| β-actin | Rabbit | Cell Signalling | 3700 | 1:2500 | 1 hour room temperature |
| Anti-Opsin Antibody, Red/Green | Rabbit | Millipore | AB5405 | 1:1000 | 4^o^C overnight |
| Anti-Opsin Antibody, Blue | Rabbit | Millipore | AB5407 | 1:1000 | 4^o^C overnight |
| Glycogen Phosphorylase | Guineapig | Custom made [1] | N/A | 1:1000 | 4^o^C overnight |
| Anti-Cone Arrestin Antibody | Rabbit | Millipore | AB15282 | 1:1000 | 4^o^C overnight |
| Goat Anti-Rabbit IgG H+L AlexaFluor 568 | Rabbit | Abcam | AB175471 | 1:500 | 2 hours room temperature |
| Goat Anti-Mouse IgG H+L AlexaFluor 568 | Mouse | ThermoFisher | AB11031 | 1:500 | 1 hour room temperature |
| Goat Anti-Guineapig IgG FITC | Guineapig | Merck | F6261 | 1:300 | 1 hour room temperature |
|  |  |  |  |  |  |


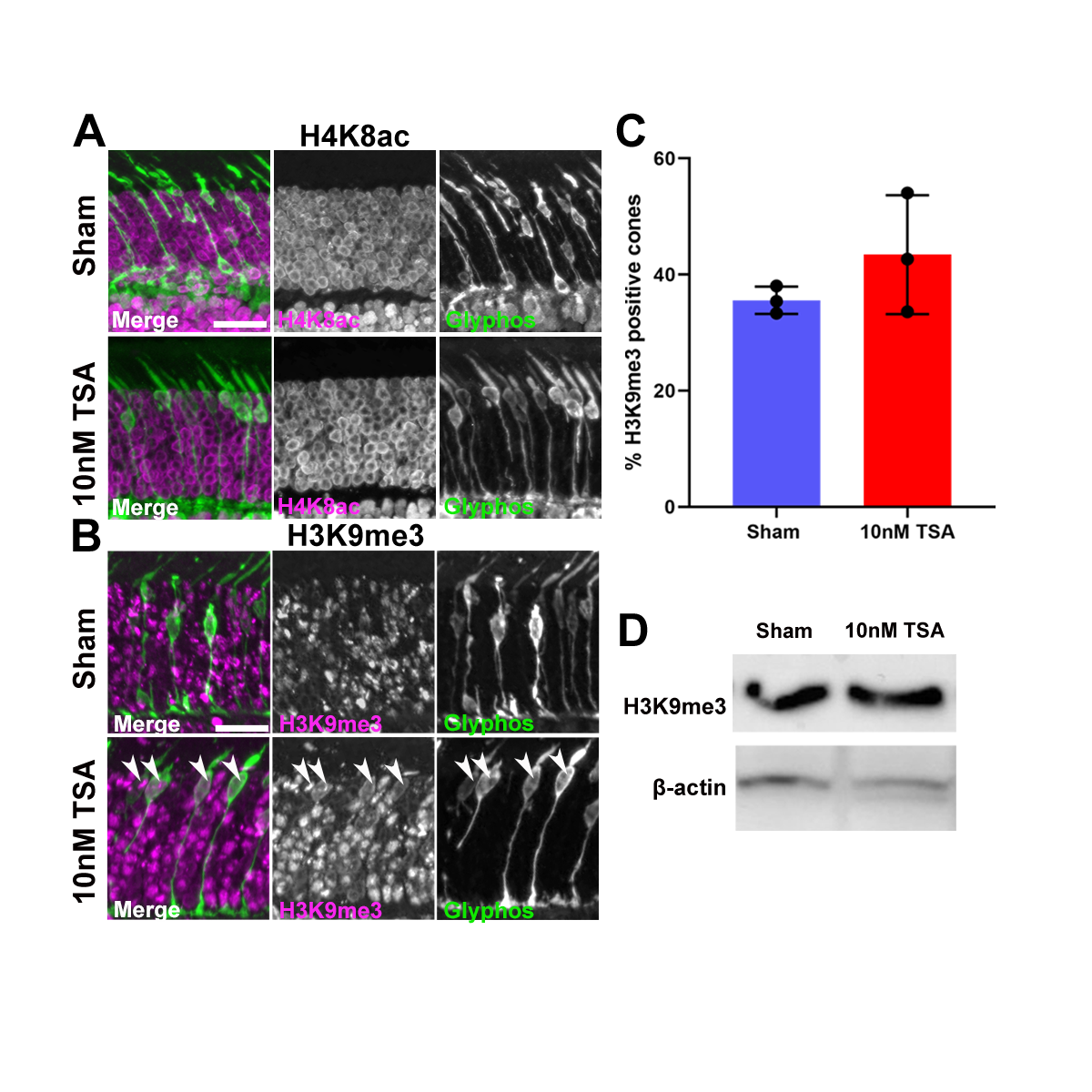


**Fig. S1 A** The immunofluorescence staining patterns with acetylated H4K8 antibody (magenta) was similar in untreated and treated *Pde6c^cpfl1^* photoreceptors following a single intravitreal TSA injection. Scale bar 20µm **B** H3K9me3 staining (magenta) was detected in both untreated and TSA-treated cones (stained in green with Glyphos antibody) denoted by arrows. Scale bar 20µm **C** No significant change was detected after quantification of the percentage of H3K9me3 positive cones in sham controls and TSA-treated *Pde6c^cpfl1^* mice. Welsh’s T-test, n=3, *P*>0.05 **D** A Western blot of the whole retina revealed no change in H3K9me3 protein abundance after treatment with 10nM TSA. β-actin was used as the housekeeping control


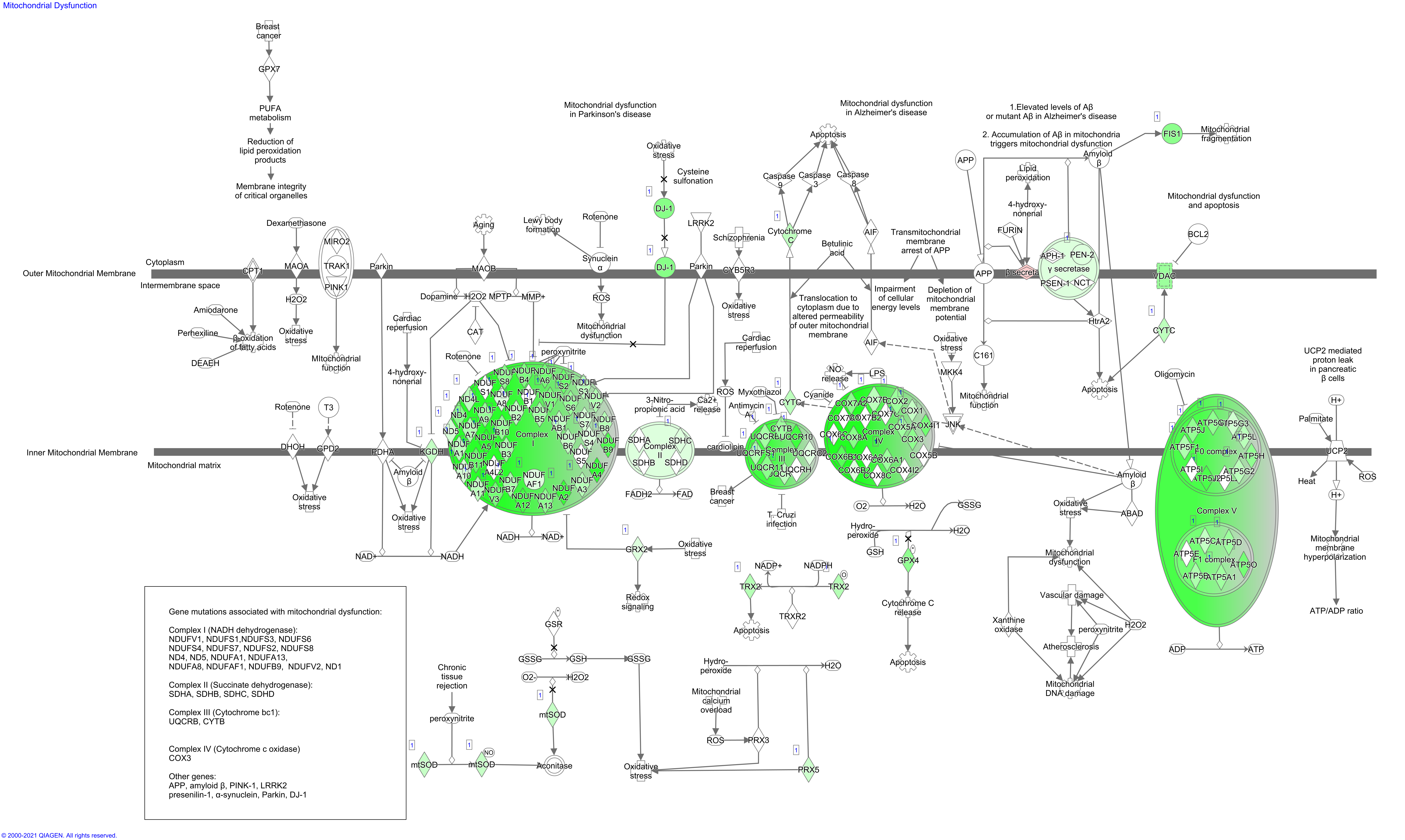


**B**

**GSK-J4 treated**


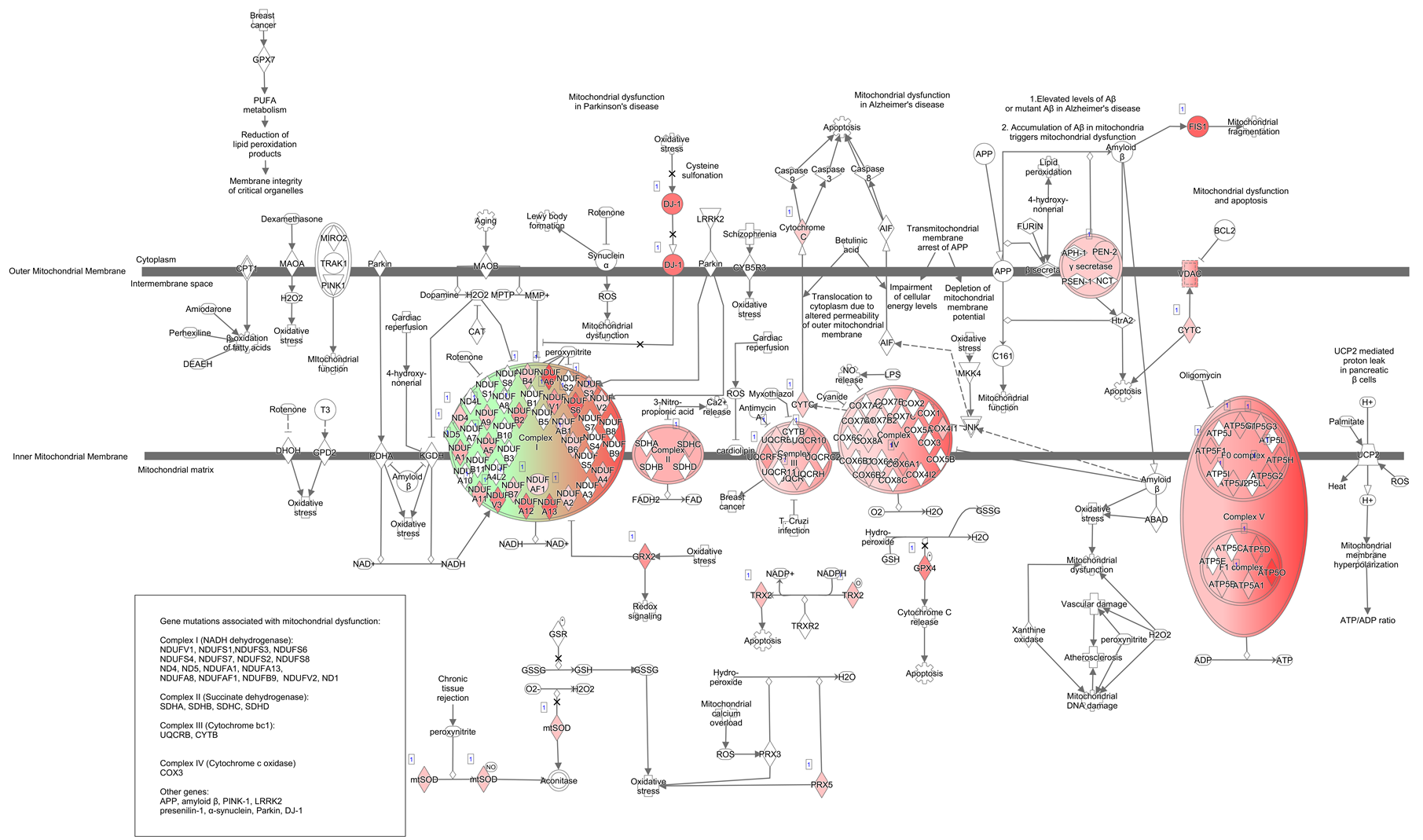


**A**

**Uninjected**

**Fig. S2** Schematic diagrams of the mitochondrial dysfunction pathway in **A** uninjected and **B** GSK-J4 treated *Pde6c.*GFP cones, with expression levels of genes overlaid. After treatment with GSK-J4, we observed a significant change in the expression profile, with downregulation of key genes in complexes I-V, which are responsible for oxidative phosphorylation. Diagram downloaded from QIAGEN Ingenuity Pathway Analysis, genes shown in the diagram have >0.3 absolute log fold change, and an unadjusted *P*<0.01. Green downregulation; red upregulation

**GSK-J4 treated**

**
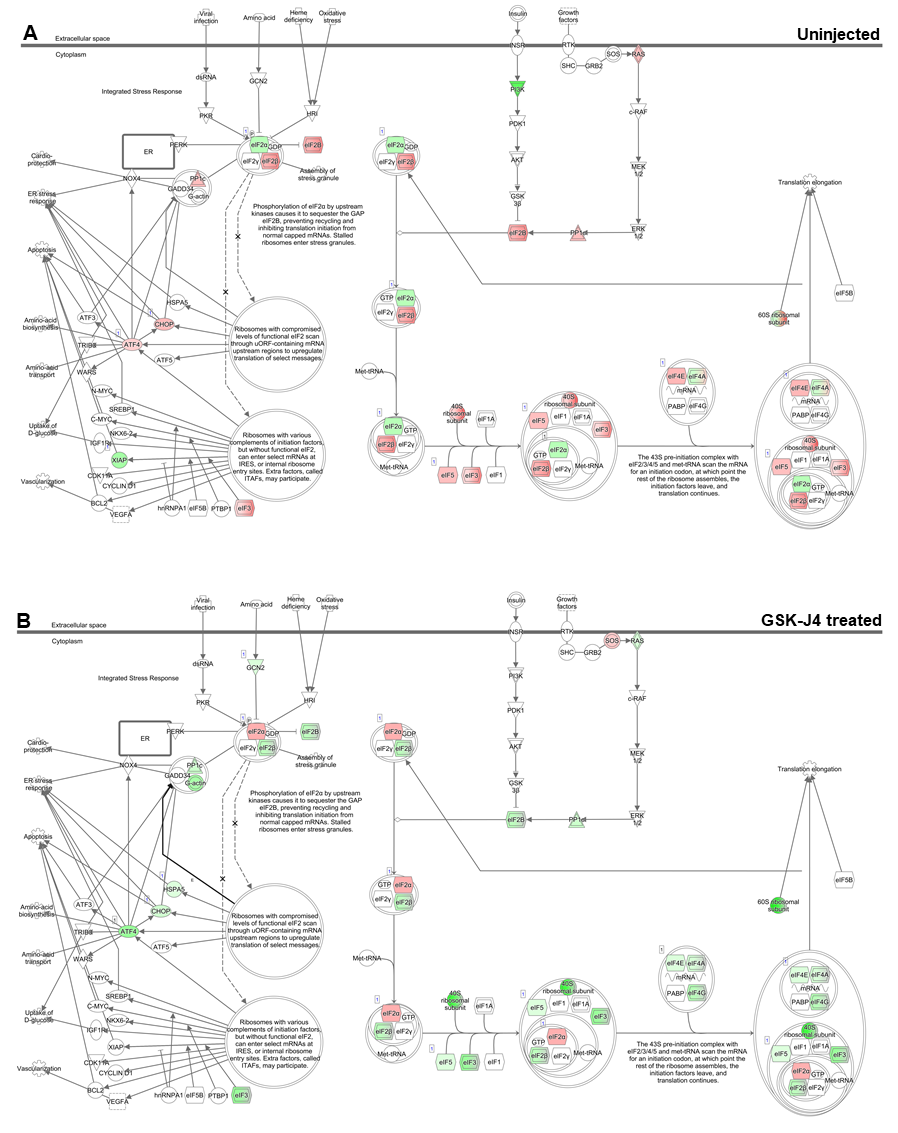
**

**Fig. S3** Schematic diagrams of the EIF2 signaling pathway in **A** uninjected and **B** GSK-J4 treated *Pde6c.*GFP cones, with expression levels of genes overlaid. After treatment with GSK-J4, we noted downregulation of the CHOP cell death pathway, and an overall reduction in protein production. Diagram downloaded from QIAGEN Ingenuity Pathway Analysis, genes shown in the diagram have >0.3 absolute log fold change, and an unadjusted *P*<0.01. Green downregulation; red upregulation


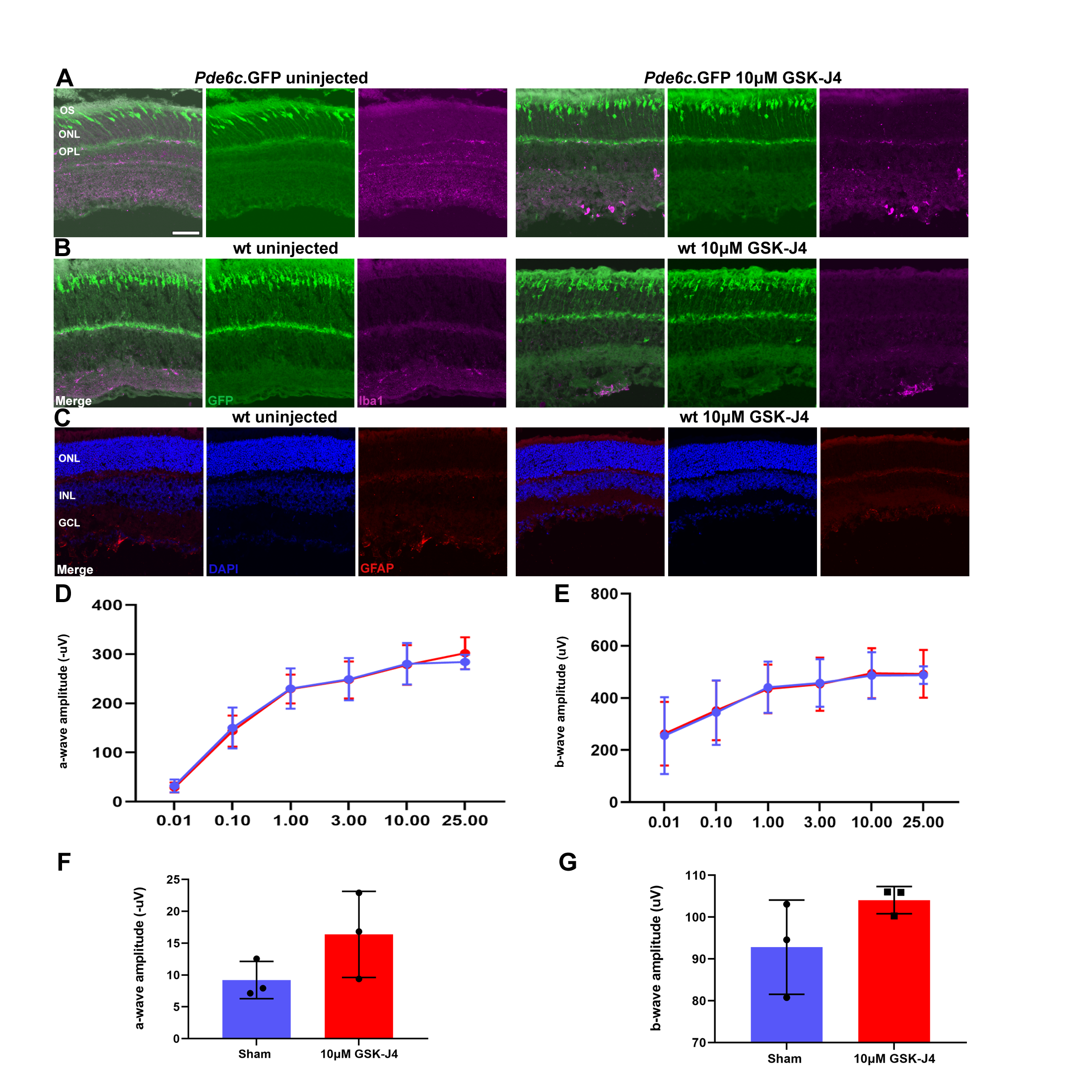


**Fig. S4 A** Iba1 microglial staining (magenta) in *Pde6c.*GFP mice injected with GSK-J4 appeared slightly increased, but with no change in morphology (activation) of microglial cells or migration to the outer nuclear layer or subretinal space. Scale bar 50µm **B** Similarly, in wt mice treated with 10µM GSK-J4, no microglia activation of migration to the ONL was evident **C** GFAP (activated Müller Glia; red) staining in wt retinae was not increased after treatment with GSK-J4 **D, E** Scotopic ERG recordings in wt mice treated with GSK-J4 revealed no change in a- or b-wave amplitude. Two-way ANOVA, n=3, *P*>0.05 **F, G** After the treatment with GSK-J4, photopic ERG responses were comparable to sham controls. Bar graphs show the a- and b-wave amplitude with a stimulus intensity of 10 cd.s.m^-2^. Welch’s T-test, n=3, *P*>0.05

[1] Pfeiffer-Guglielmi B, Fleckenstein B, Jung G and Hamprecht B (2003) Immunocytochemical localization of glycogen phosphorylase isozymes in rat nervous tissues by using isozyme-specific antibodies. J Neurochem 85:73-81. 10.1046/j.1471-4159.2003.01644.x.
